# “Electron transport chain interference” strategy of amplified bacterial ferroptosis and defect-engineered nanozyme for diabetic wound healing

**DOI:** 10.7150/thno.121636

**Published:** 2026-01-01

**Authors:** Yanlan Xie, Huan Wang, Yalan Wang, Jiajie Liu, Jinming Tong, Tao Wu, Li Yin, Chuan Zhang, Long Zhao, Yuan Yong

**Affiliations:** 1 Key Laboratory of Pollution Control Chemistry and Environmental Functional Materials for Qinghai-Tibet Plateau of the National Ethnic Affairs Commission, School of Chemistry and Environment, Southwest Minzu University, Chengdu 610041, China.; 2 Nanomedicine Innovation Research and Transformation Institute, Affiliated Hospital of North Sichuan Medical College, Nanchong 637000, China.; 3 Biotechnology Innovation Drug Application and Transformation Key Laboratory of Sichuan Province, North Sichuan Medical College, Nanchong 637000, China.; 4 Department of Neurosurgery, Affiliated Hospital of North Sichuan Medical College, Nanchong 637000, China.

**Keywords:** defect engineering, nanozyme, ferroptosis, bacterial electron transport chain, diabetic wound healing

## Abstract

**Background:** The pathological hyperglycemic microenvironment in diabetic wounds increases susceptibility to bacterial infections and impairs wound healing. However, despite certain advancements in conventional clinical treatments, the pathological issues have not yet been fundamentally resolved. The mechanism that amplifies ferroptosis through disruption of the bacterial electron transport chain (ETC) results in effective bacterial eradication and facilitates wound healing, thereby offering novel therapeutic potential for the management of diabetic wound infections.

**Methods:** This work designs a multi-enzyme-mimicking Fe-WS_2_@GOx nanozymes by loading glucose oxidase (GOx) onto defect-engineered Fe-WS_2_, which disrupts the bacterial ETC and induces ferroptosis, thereby accelerating diabetic wound healing.

**Results:** During the bacterial infection stage, the Fe-WS_2_@GOx nanozymes with abundant sulfur vacancies can simultaneously mitigate hyperglycemic and alleviate the hypoxic microenvironment. This is achieved through continuously producing substantial amounts of reactive oxygen species (ROS), resulting in endogenous glucose consumption, promoting cyclic accumulation of H_2_O_2_, and ensuring a sustained oxygen supply. Meanwhile, the generated ROS interferes with the bacterial ETC, impedes bacterial energy metabolism and inhibits biosynthesis, ultimately leading to bacterial death. More importantly, at the new tissue proliferation stage, Fe-WS_2_@GOx can promote wound angiogenesis and tissue regeneration by macrophage immunomodulatory effect.

**Conclusions:** Therefore, this study provides a new paradigm strategy for diabetic wound infections therapy by “electron transport chain interference” amplified bacterial ferroptosis.

## Introduction

Diabetic wounds are characterized by hyperglycemia, which increases the opportunity for bacterial colonization at the lesion site, making the wounds more susceptible to infection 1, 2. Currently, common treatment methods include antibiotic therapy, debridement, and blood glucose control 3, 4. Although these approaches have achieved some clinical progress, they fail to address the underlying pathological issues of the wounds during patient treatment. Nanozymes, due to their unique enzyme-like catalytic activity and tunable physicochemical properties, have demonstrated great potential in the field of anti-infection 5. However, single nanozyme therapy still faces challenges in improving catalytic efficiency and ensuring biocompatibility 6.

Nanozymes require high catalytic activity to effectively exert their desired antibacterial effects. To enhance the catalytic performance of nanozymes, defect engineering has been demonstrated as an effective strategy to modulate their structure and optimize functionality 7, 8. It has been previously reported that the construction of defects on the surface of nanozymes, particularly through the incorporation of transition metals (e.g., Fe, Mn, Co), can not only enhance the catalytic activity of nanozymes but also optimize the binding energy of the reaction intermediates and lower the energy barrier 9-11. In addition, numerous defects disrupt the crystal structure of the substrate, leading to an increase in active sites and facilitating electron transfer between the substrate and nearby electrons 12. Notably, sulfur vacancies serve as novel tools for regulating free radicals by reconfiguring the electronic structure and enabling rational adsorption of reaction intermediates on the surface of the nanozymes 13-15. After NIR irradiation, sulfur vacancies promote the regulation of electronic and energy band structures, thereby enhancing the efficiency of ROS generation 16-18. Based on these findings, we propose the use of defect engineering and NIR to design nanozymes containing sulfur vacancies and cascade catalytic capabilities, which are expected to produce substantial amounts of ROS and enhance the antimicrobial activity. However, a single nanozyme with defect engineering may experience lattice distortion and phase transition, which could adversely affect its catalytic activity and ROS generation efficiency 19.

With further research, scholars have discovered that other therapeutic approaches can compensate for the inherent limitations of catalytic therapy. Ferroptosis, a regulated form of cell death dependent on iron ions, initially garnered significant interest within tumor research; however, recent studies have revealed that it is also holds particular promise in the field of antimicrobial therapy 20, 21. In bacteria, ferroptosis is co-induced through elevated uptake of unstable iron ions and enhanced lipid peroxidation by the Fenton reaction 22. Meanwhile, the bacterial respiratory chain is also affected by ferroptosis 23. The bacterial ETC, a core component of energy metabolism, is primarily located on the cell membrane 24. It facilitates ATP production through redox reactions and helps maintain cellular reducing power (NAD⁺/NADH balance) 25. During ferroptosis, the generated ROS can oxidize cysteine residues in active sites of ETC complexes (e.g., Complex I, II, III), leading to conformational changes or functional impairment 26, 27. Disruption of the ETC prevents the formation of the proton gradient, compromises ATP synthase function, and blocks bacterial energy metabolism. This process starves bacterial cells, ultimately leading to DNA and membrane damage that induces bacterial death. Therefore, targeting the bacterial ETC through ferroptosis by generating abundant ROS alters biofilm energy metabolism and redox homeostasis, representing a highly promising antibacterial strategy. In reality, the in situ catalytic capacity of enzymes is limited, hindering the execution of such complex biomedical reactions. Fortunately, GOx, an oxygen-demanding dehydrogenase, can consume glucose within the diabetic wound microenvironment to promote cyclic accumulation of endogenous H_2_O_2_, thereby reducing hyperglycemia, facilitating wound healing, and overcoming limitations in localized catalytic activity 28. Thus, developing GOx-loaded defect-engineered nanozymes to induce bacterial ferroptosis is particularly important for compensating the constrained catalytic performance of single defect-engineered nanozymes.

**Scheme 1:**
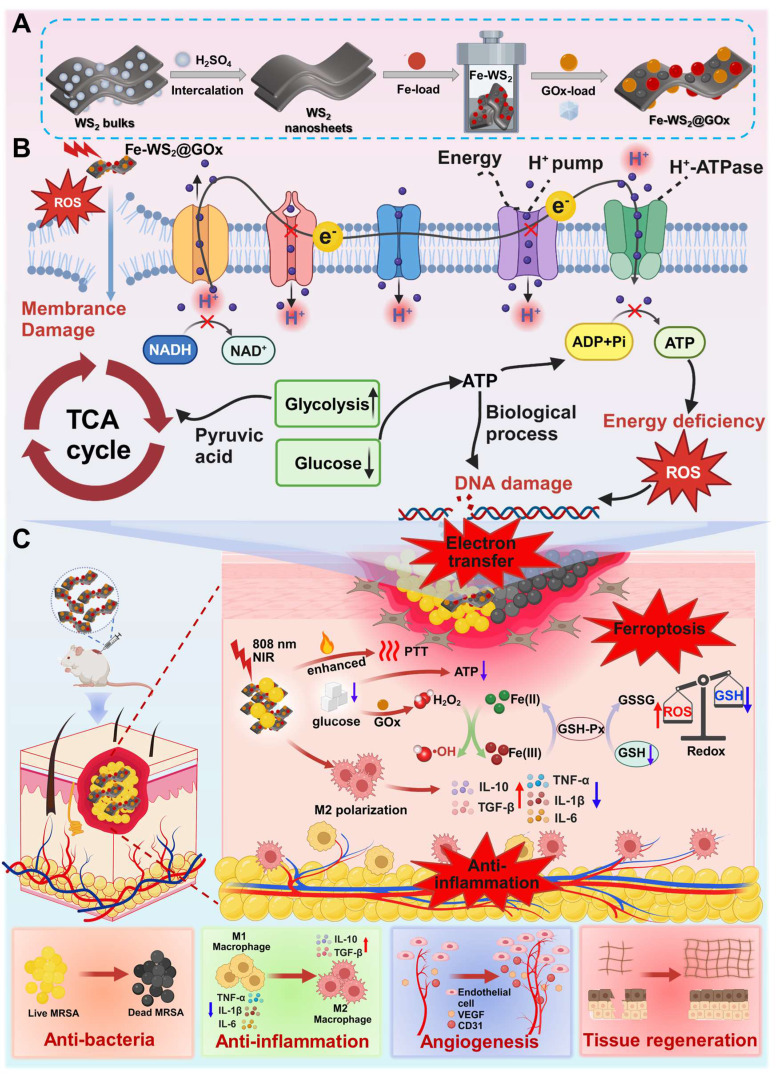
Schematic illustration of (A) the preparation process of Fe-WS_2_@GOx, (B) its mechanism of disrupting the bacterial ETC in the bacterial microenvironment and synergistically amplifying ferroptosis, and (C) its role in promoting diabetic wound healing.

In this study, we developed Fe-WS_2_@GOx nanozymes via defective engineering, which exhibit an enzyme cascade catalytic effect capable of interfering with the bacterial ETC and inducing bacterial ferroptosis. This leads to notable antimicrobial and anti-inflammatory effects, thereby accelerating diabetic wounds healing. In this work, iron substitution was employed to design nanozymes with a defective structure, resulting in the formation of abundant sulfur vacancies (Scheme 1). Under NIR, the sulfur vacancies in Fe-WS_2_@GOx nanozymes enhance ROS generation efficiency by modulating the electronic and band structures. Once internalized by bacteria, the defect-engineered Fe-WS_2_@GOx nanozymes simultaneously mitigate hyperglycemic and alleviate the hypoxic microenvironment through continuous ROS production, endogenous glucose and GSH consumption, cyclic accumulation of H_2_O_2_ and O_2_ sustainable supplement. These processes trigger lipid peroxidation and ultimately lead to ferroptosis. In addition, elevated ROS levels inhibit key enzymes in the bacterial respiratory chain, while GOx also consumes the bacteria's energy, impairing ATP synthesis and interfering ETC function, resulting in bacterial death. Furthermore, *in vitro* and *in vivo* experiments demonstrated that Fe-WS_2_@GOx can promote the polarization of pro-inflammatory M1-type macrophages to anti-inflammatory M2-type macrophages, enhancing local immunomodulatory activity and facilitating diabetic wound healing. Therefore, the defect-engineered Fe-WS_2_@GOx nanozymes represent a novel therapeutic strategy that utilizes ROS-mediated disruption of the bacterial ETC and induction of ferroptosis, while simultaneously modulating the immune microenvironment to improve diabetic wound healing.

## Experimental Methods

### Materials

Tungsten disulfide (WS_2_), 3,3',5,5'-tetramethylbenzidine (TMB) and glutathione (GSH) were purchased from Alpha (Shanghai) Essar Reagent Co. Methyl blue (MB) and o-phenanthroline hydrochloride monohydrate were purchased from Aladdin (Shanghai) Reagent Co. Concentrated sulfuric acid (H_2_SO_4_), potassium bromide (KBr), ferric chloride hexahydrate (FeCl_3_∙6H_2_O) and ascorbic acid were purchased from Chengdu Cologne Chemical Co. Hydrogen peroxide (H_2_O_2_, 30%) was purchased from Chengdu Jinshan Chemical Reagent Co. Glucose oxidase (GOx) was purchased from Shanghai Yuan Ye Biotechnology Co. Tryptic Soy Peptone Liquid Medium (TSB) and Soy Casein Agar Medium (TSA) were purchased from Beijing Auboxin Biotechnology Co. 2',7'-Dichlorodihydrofluorescein diacetate (DCFH-DA) was purchased from Biyuntian Biotechnology Co. BCA Protein Assay Kit was purchased from Beijing Kulabo Science and Technology Co. ATP assay kit was purchased from Solarbio Life Sciences. YTO 9/PI Double Staining Kit for Live/Dead Bacteria was purchased from MKbio. All reagents were used as received. All aqueous solutions were prepared with deionized water.

### Characterization

Transmission electron microscope (Tecnai G2 F30, FEI, USA) was used to observe the morphology of WS_2_ and Fe-WS_2_. The UV-visible absorption spectra of the substances were determined by a UV spectrophotometer (UV-6100, Mapada, Shanghai, China). A nanopotentiostat (Zetasizer Nano ZS, Malvern Instruments Ltd., UK) was used to measure particle size and zeta potential. A Fourier transform infrared spectrometer (IR 200, Thermo Fisher Scientific) and an X-ray photoelectron spectrometer (K-alpha, Thermo Fisher Scientific) were used to characterize valence bond compositions and elemental valence distributions.

### Preparation of WS_2_, Fe-WS_2_ and Fe-WS_2_@GOx

WS_2_ nanozymes were prepared using a concentrated sulfuric acid pickling intercalation method. Specifically, commercially obtained WS_2_ powder was milled with a vertical planetary ball mill for 6 h. Next, 60 mL of concentrated sulfuric acid was added to 60 mg of milled WS_2_ powder, and the mixture was heated in an oil bath at 90 °C for 24 h. After cooling, the product was washed via centrifugation. Then, the product was dispersed in deionized water and sonicated with a cell crusher for 8 h. The sonicated product was again washed by centrifugation and finally collected for lyophilization.

Fe-WS_2_ nanozymes were synthesized via a hydrothermal method. Specifically, 15 mg of WS_2_ nanozymes, 15 mg of FeCl_3_·6H_2_O and 15 mg of ascorbic acid were added to 30 mL of deionized water a 1:1:1 mass ratio. The mixture was stirred continuously at room temperatur for 1 h using a magnetic stirrer. Then, 10 mg of NaHCO_3_ was added, and stirring was continued for 20 min. The resulting mixture was transferred to a reactor and reacted for 6 h at 150 °C. After the reaction was allowed to cool to room temperature, the product was washed by centrifugation and the solid was collected.

Subsequently, Fe-WS_2_ nanozymes and glucose oxidase were added according to a mass ratio of 1:2, stirred in an ice bath for 24 h, and then centrifuged and washed to obtain iron-doped WS_2_ nanozymes loaded with glucose oxidase.

### Peroxidase-like activity

Measurements were conducted using MB as a probe. Specifically, H_2_O, H_2_O_2_, Fe-WS_2_@GOx and Fe-WS_2_@GOx+H_2_O_2_ were each introduced into MB and allowed to react for 15 min. The absorbance at the characteristic peak (λ = 610 nm) was measured using a UV spectrophotometer.

### Glutathione-like peroxidase activity

Measurements were performed using DNTB as a probe. Specifically, a bicarbonate buffer solution at 0.1 mM GSH (0.5×10^-3^ M, pH 8) was mixed with Fe-WS_2_@GOx at concentrations of 50, 100 and 200 μg/mL, respectively. As the reaction progressed, 1980 μL of Tris-HCl (0.5×10^-3^ M, pH 8) solution and 20 μL of 10 mM DTNB (0.5×10^-3^ M, pH 8) solution were added at different times. The absorbance at the characteristic peak (λ = 412 nm) was measured using a UV spectrophotometer.

### Fe^2+^ release

To measure GSH-triggered Fe^2+^ release in microbial environments, Fe-WS_2_@GOx (2 mg) was dispersed in solutions with and without GSH (0.2 mM), respectively. The suspension was dialyzed in a buffer medium for 24 h (12 kDa MW). At selected time intervals, 1 mL of each dialysate was withdrawn and replaced with an equal volume of fresh buffer medium. The released Fe^2+^ was collected and reacted with o-phenanthroline solution for 15 min. The absorbance at the characteristic peak (λ = 512 nm) was measured using a UV spectrophotometer.

### DFT calculation

All theoretical calculations in this study were conducted within the framework of density functional theory (DFT) employing the Vienna Atomic Force Simulation Package (VASP). The core electron interactions were described using the spin-polarized projected augmented wave (PAW) method.

### NIR enhanced ROS

To investigate the photo-enhanced catalytic performance of Fe-WS_2_ nanozymes in H_2_O_2_-mediated ROS generation, POD-like activity was assessed using TMB as the chromogenic substrate. The assays were conducted under NIR irradiation or in the absence of NIR, with H_2_O_2_ concentrations of 5 mM, 8 mM, and 10 mM. Additionally, the catalytic activity was evaluated at different NIR power densities (0 W/cm^2^, 0.2 W/cm^2^, 0.5 W/cm^2^ and 1.0 W/cm^2^).

### *In vitro* antimicrobial activity

The antimicrobial activity of Fe-WS_2_@GOx was evaluated using the plate count method, live/dead staining, scanning electron microscopy, and crystal violet staining. In this study, methicillin-resistant MRSA and *E. coli* were used as models for Gram-positive bacteria and Gram-negative bacteria, respectively. After different treatments: PBS, NIR, WS_2_, WS_2_+NIR, Fe-WS_2_, Fe-WS_2_+NIR, Fe-WS_2_@GOx and Fe-WS_2_@GOx+NIR were incubated for 4 h at 37 °C with shaking. The bacterial suspensions were subsequently irradiated with 808 nm laser (1 W/cm^2^, 10 min). Subsequently, 100 μL of the bacterial suspensions were transferred to agar plates and photographed and counted for the number of bacteria after 12 h of incubation at 37 °C. For fluorescent staining, acridine orange and ethidium bromide (AO-EB) were applied to all treatment groups. The stains were at 4 °C for 15 min, and a drop of each bacterial suspension was placed on a slide and observed under an inverted fluorescence microscope. For scanning electron microscopy, bacteria subjected to different treatments were fixed with 2.5% glutaraldehyde for 10 h, centrifuged (3000 rpm, 10 min), and washed three times with saline. Then, it was sequentially eluted using 30%, 50%, 70%, 90% and 100% ethanol for 15 min. Finally, the bacterial solution was dropped on slides, which were dried and characterized by scanning electron microscopy. For the crystal violet staining experiments, 100 μL of diluted *E. coli* and MRSA suspensions were mixed with 100 μL of TSB and added to a 96-well plate, followed by incubation at 37 °C for 48 h. The medium was replaced every 12 h. After incubation, bacterial biofilms from each treatment group were fixed with anhydrous ethanol and stained with crystal violet. The OD_590_ value was measured using an enzyme marker and the value and color were recorded.

### *In vitro* biofilm disruption

The ability of Fe-WS_2_@GOx nanozymes to remove bacterial biofilm was tested against by crystal violet staining. Bacteria were introduced into 24-well plates and incubated for 3 days. After aspiration of the culture medium, the resulting biofilms were subjected to different treatments accordingly. The treated plates were then incubated at 37 °C for 12 h, followed by irradiated with 808 nm laser (1 W/cm^2^). Subsequently, crystal violet dye was added and incubated at room temperature for 30 min. The biofilms were washed with PBS to remove the excess dye and air-dried naturally at room temperature. The well plates were placed in an enzyme marker to detect the OD_590_ absorbance and photographs were taken to record the color of each well.

### Respiratory chain complex activity detection

For mitochondrial respiratory chain complex analysis, an assay kit (Beijing Solarbio Science & Technology Co., Ltd.) was employed. Bacteria were divided into four groups based on treatment: Control, NIR, Fe-WS_2_@GOx and Fe-WS_2_@GOx+NIR. The procedures were conducted in accordance with the manufacturer's instructions. Absorbance values for the different complexes were determined using a microplate reader.

### mRNA sequencing analysis

*E. Coli* was cultured in LB medium at 37 °C for 12 h and divided into three groups based on different treatment methods: Control, Fe-WS_2_@GOx and Fe-WS_2_@GOx+NIR. The bacterial culture was centrifuged to collect the bacterial cells, discard the supernatant, and obtain the bacterial pellet. Subsequently, grind the bacteria thoroughly in a liquid nitrogen environment, then use the TRIzol reagent kit to extract total RNA from the three groups of *E. coli* for RNA sequencing analysis. Next, the quality and integrity of total RNA were assessed using agarose gel electrophoresis, Nanodrop micro-spectrophotometer detection, and Agilent 2100 detection. The total RNA was fragmented into approximately 200 nt fragments following ribosomal RNA removal. Then, dNTPs were used with DNA polymerase I to synthesize the second strand of cDNA. In addition, selected cDNA fragments were amplified and sequenced using the Illumina HiSeq2500 platform. Finally, differences among the three groups of genes were further analyzed using KEGG and GO enrichment analysis.

### Cytotoxicity assay

The cytotoxicity of the materials was evaluated by CCK-8 assay. L929 cells were seeded into 96-well plates at 8×10^3^ per well and incubated overnight to facilitate cell attachment. Different concentrations of the materials were added and incubated for 12 h. Subsequently, the cells were washed twice with PBS. Finally, after staining with CCK-8 dye for 1 h, the absorbance of each well at 450 nm was measured using a microplate reader.

### Hemolysis assay

In the hemolysis assay, blood was collected from mouse eyeballs and transferred into anticoagulant tubes. The supernatant was discarded by centrifugation (5000 rpm, 10 min). The erythrocytes were washed twice with saline to prepare a 2% erythrocyte suspension. An appropriate amount of the test material was added to the erythrocyte suspension and incubated at 37 °C for 3 h. Saline and ultrapure water were used as the negative and positive controls, respectively. After incubation, each sample was centrifuged (5000 rpm, 10 min) and 100 μL of supernatant was transferred to a 96-well plate. The absorbance at 450 nm was measured using a microplate reader. The hemolysis ratio was calculated as follows:

Hemolysis ratio (%) = [Experiment group(OD_545_) - negative group(OD_545_)] / [positive group(OD_545_) - negative group(OD_545_)] × 100%

### Macrophage polarization

RAW264.7 was incubated with DMEM medium (10% FBS, 1% double antibody) at 37 °C, 5% CO_2_. 1×10^5^ RAW264.7 per well was accessed into a 6-well plate and incubated for 24 h. RAW264.7 was stimulated to M1 phenotype by treatment with 0.1 μg/mL LPS. After different treatments (Control, NIR, Fe-WS_2_@GOx, Fe-WS_2_@GOx+NIR) for 24 h. The cells were then co-incubated with antibodies against anti-CD80 and anti-CD206, respectively, and detected by flow cytometry after 30 min of incubation.

### *In vivo* diabetic wound healing assessment

All animal experimental protocols and human participant procedures were approved by the Academic Ethics Committee of Southwest Minzu University, Sichuan Province (SMU-202501127). Female Sprague-Dawley mice (204-208 g) were selected and purchased from Chengdu Dossy Laboratory Animal Co. Type 2 diabetes was induced via intraperitoneal injection of a preformed streptozotocin (STZ) solution at a dosage of 50 mg/kg. One week after the injection, mice exhibiting blood glucose levels ≥16.7 mmol/L in two consecutive measurements were deemed diabetic. These diabetic mice were randomly divided into eight groups: control, NIR, WS_2_, WS_2_+NIR, Fe-WS_2_, Fe-WS_2_+NIR, Fe-WS_2_@GOx and Fe-WS_2_@GOx+NIR, with three mice in each group. Subsequently, the dorsal wound surface was infected with MRSA suspension (1×10^6^ CFU/mL). After 24 h, 5 μL of a different material (50 μg/mL) was dropped into the subcutaneous pustules at the infection site in each group. The wounds were then irradiated using a near-infrared light source at a power density of 1 W/cm^2^ for 10 min. Wound size and body weight were recorded on days 1, 3, 6, and 12 post-treatment. After 12 days of treatment, the mice were euthanized, and blood samples were collected for hematological analysis. Heart, liver, spleen, lung, kidney, and wound tissues were collected, fixed in 4% paraformaldehyde solution, and stained with H&E for histological analysis.

### Immunofluorescence and immunohistochemical analysis

The wound tissue was fixed with 4% paraformaldehyde solution and embedded in paraffin for sectioning. Subsequently, the sections were co-incubated with primary and secondary antibodies together with DAPI, and observed under a fluorescence microscope. Wound tissue angiogenesis and wound healing ability were observed using CD31, Col 1 and MPO antibodies. The expression of anti-inflammatory and pro-inflammatory cytokines was detected using antibodies against TNF-α, IL-6, IL-1β, TGF β1 and IL-10 antibodies. As well as CD80 and CD206 antibodies to detect macrophage polarization. Finally, ferroptosis was detected with GPX4 antibody.

### Statistical analysis

Origin 2022 software was used to analyze the data. All experiments were performed with at least three measurements and results were expressed as mean ± standard deviation (SD). Asterisks indicate significant differences (*P ≤ 0.05, **P ≤ 0.01, ***P ≤ 0.001), analyzed by Student's t-test.

## Results and Discussion

### Preparation and characterization of Fe-WS_2_@GOx

**Figure 1:**
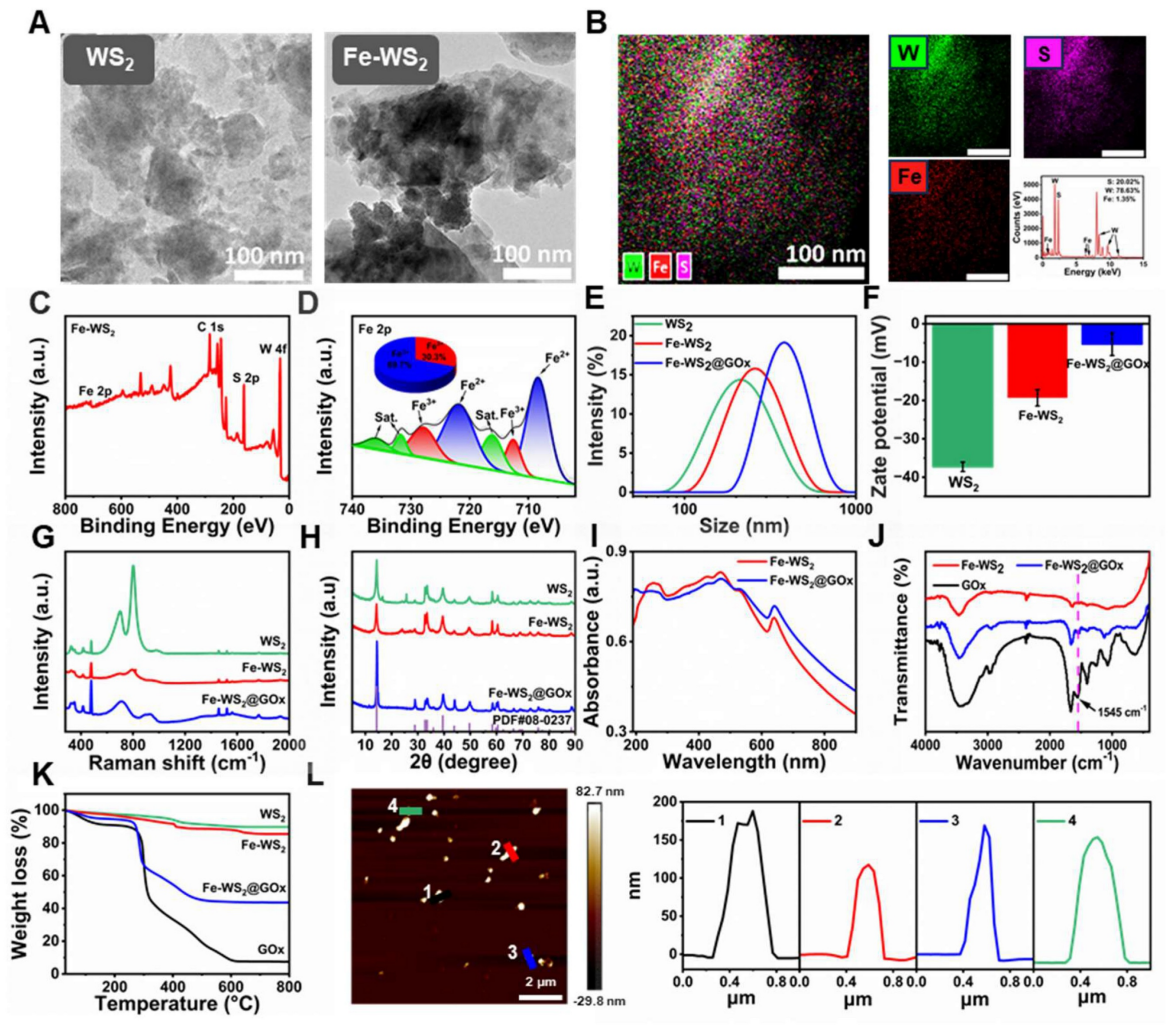
** Characterization of Fe-WS_2_@GOx nanozymes.** (A) TEM images of WS_2_, Fe-WS_2_ nanozymes. (B) EDS of Fe-WS_2_. (C) XPS full spectrum of Fe-WS_2_. (D) Fine Fe 2p XPS spectrum of Fe-WS_2_. (E-H) DLS, zeta potential, Raman and XRD of WS_2_, Fe-WS_2_ and Fe-WS_2_@GOx. (I) UV spectra of Fe-WS_2_ and Fe-WS_2_@GOx. (J) FT-IR spectra of Fe-WS_2_ and Fe-WS_2_@GOx. (K) TGA of WS_2_, Fe-WS_2_ and Fe-WS_2_@GOx. (L) AFM image of Fe-WS_2_@GOx.

Fe-WS_2_@GOx with engineering defect was synthesized by a series of acid-washing, hydrothermal and ice-bath methods (Scheme 1). The transmission electron microscope (TEM) images of WS_2_ and Fe-WS_2_ nanozymes were first shown in Figure 1A, both of which were composed of lamellar structure with excellent monodispersity, and mean diameters of approximately 200 and 300 nm, respectively. X-ray energy dispersive spectroscopy (EDS) and XPS spectra were shown the well-proportioned distribution of Fe, W, and S in Fe-WS_2_, indicating that Fe atoms were highly dispersed throughout the nanozymes, which provided solid evidence for the successful synthesis of Fe-WS_2_ (Figure 1B-C). Subsequently, the valence states of Fe, W, and S were further analyzed (Figure 1D and S1). The Fe2p spectrum was shown that the principal peaks at approximately 721 and 709 eV corresponded to Fe^2+^ of Fe-WS_2_. And the other two peaks at approximately 728 and 712 eV were attributed to Fe^3+^. The coexistence of mixed Fe^2+^/Fe^3+^ valence states demonstrated its excellent redox properties. Furthermore, the successful synthesis of Fe-WS_2_@GOx was also demonstrated by a series of tests including dynamic light scattering (DLS), zeta potential and Raman (Figure 1E-G). Thereafter, the crystal structures of biocatalysts have been identified by X-ray diffraction (XRD). The diffraction peaks of all samples can be well indexed to WS_2_ (PDF#08-0237), and no diffraction peaks associated with crystalline Fe species can be detected in both Fe-WS_2_ and Fe-WS_2_@GOx, indicating that Fe only existed in substitutional form (Figure 1H). Moreover, the ultraviolet-visible (UV-vis) spectra shown strong absorption of Fe-WS_2_@GOx in the near-infrared region at 700-900 nm (NIR-I), which suggested an excellent photothermal conversion capability (Figure 1I). In the Fourier transform infrared spectroscopy (FT-IR) diagram, a characteristic peak of Fe-WS_2_@GOx was present at 1545 cm^-1^, which was the amide II band of GOx, indicating the successful loading of GOx (Figure 1J). Afterward, the thermogravimetric analysis (TGA) of Fe-WS_2_@GOx was carried out, revealing a GOx loading capacity of 9.71% and verifying the successful loading of the natural enzyme as a drug (Figure 1K). In addition, atomic force microscopy (AFM) results shown that the Fe-WS_2_@GOx nanozymes were uniform with a thickness of about 120-180 nm (Figure 1L). Therefore, it is conceivable that the defect-engineered Fe-WS_2_@GOx with Fe^2+^/Fe^3+^ mixed valence state could enhance the utilization efficiency of the active site by accelerating electron transfer, leading to an enhanced enzyme-like catalytic effect for disrupting the redox homeostasis of bacteria.

### Multi-enzyme catalytic activity and catalytic mechanism of Fe-WS_2_@GOx nanozymes

**Figure 2:**
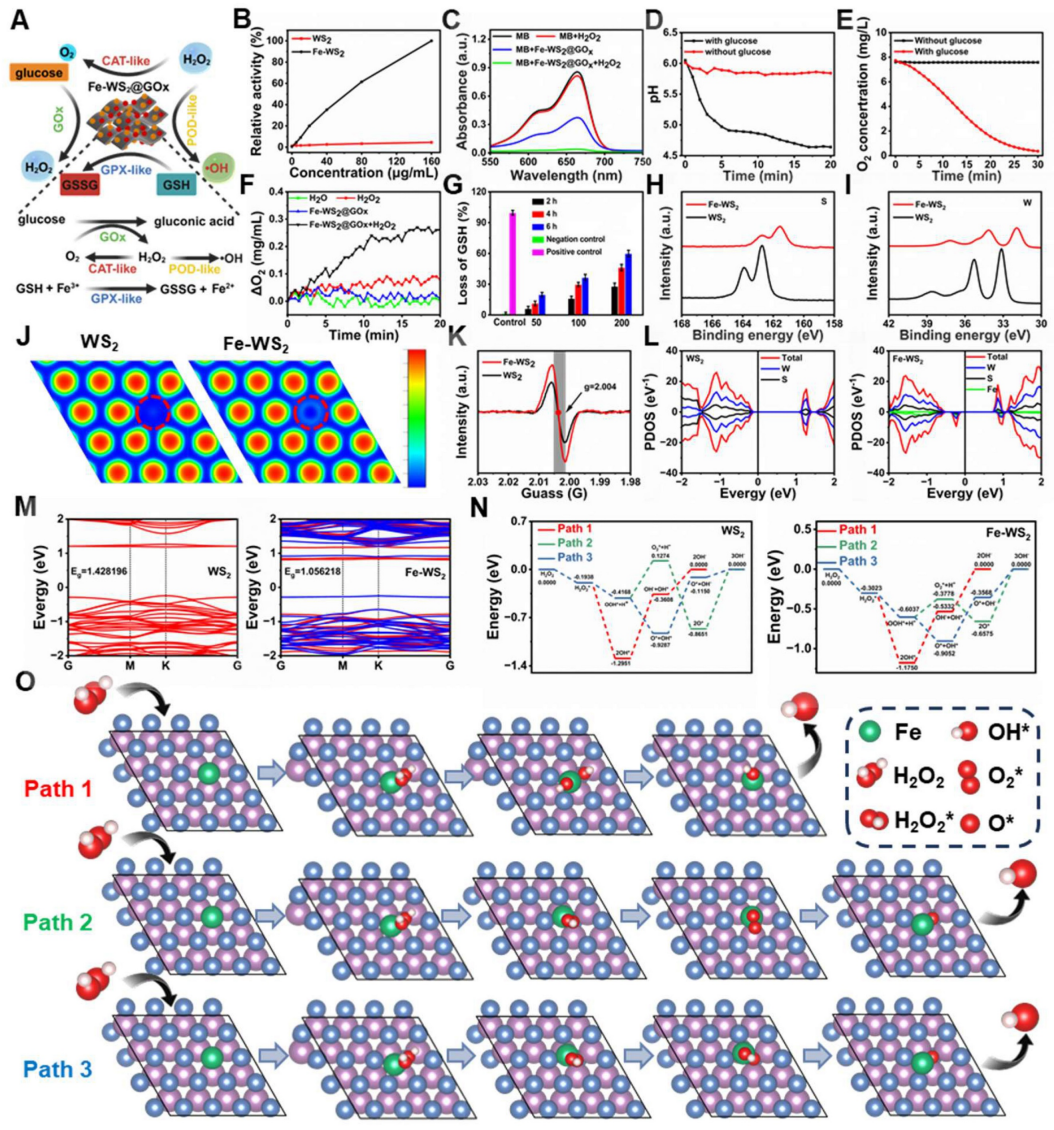
** Multi-enzyme catalytic activity and catalytic mechanism of Fe-WS_2_@GOx nanozymes.** (A) Schematic representation of the multienzyme-like activity of Fe-WS_2_@GOx. (B) Relative activities of POD-like enzyme activities of Fe-WS_2_ and WS_2_ nanozymes. (C) POD-like enzyme activity of Fe-WS_2_@GOx. Changes in (D) pH and (E) O_2_ content in the presence or absence of glucose. (F) CAT-like and (G) GSH-like enzyme activities of Fe-WS_2_@GOx. High-resolution XPS (H) S and (I) W of WS_2_ and Fe-WS_2_. (J) ELF analysis of WS_2_ and Fe-WS_2_. (K) EPR spectra of WS_2_ and Fe-WS_2_. (L) PDOS curves and (M) electronic energy band structures of WS_2_ and Fe-WS_2_ structures. (N) Gibbs free energy of WS_2_ and Fe-WS_2_. (O) The corresponding reaction pathway for H_2_O_2_-catalyzed Fe-WS_2_.

To further validate the nanozymes as an effective antimicrobial agent, we comprehensively investigated their multi-enzyme catalytic activities (Figure 2A). Before validating the activity of Fe-WS_2_@GOx nanozymes, we first compared the peroxidase (POD)-like enzyme activity of Fe-WS_2_ with that of WS_2_ to verify the superior activity after the substitution of Fe. It was found that Fe-WS_2_ exhibited stronger POD-like enzyme activity compared to WS_2_ (Figure 2B). Therefore, we hypothesized that the addition of Fe induced defective sites and enhanced electron transfer, thereby increasing the POD-like activity. The POD-like activity of Fe-WS_2_@GOx nanozymes was estimated using methylene blue (MB) as an indicator. As shown in Figure 2C, there was no significant change in the absorption peaks of MB or H_2_O_2_ alone, whereas MB was almost completely degraded as observed from the UV absorption values for the Fe-WS_2_@GOx+H_2_O_2_ group, revealing the excellent POD-like enzyme activity of Fe-WS_2_@GOx. In addition, the absorbance of MB gradually decreased with the reaction time, which was attributed to the generation of ·OH (Figure S2). As an aerobic dehydrogenase, GOx is highly specific for glucose and catalyzes the production of H_2_O_2_ and gluconic acid from glucose, thus blocking the energy supply of bacteria 29. We indirectly assessed the glucose oxidase (GOx)-like activity of Fe-WS_2_@GOx by MB degradation assay. As shown in Figure S3, there was no GOx-like activity in the presence of glucose or MB, whereas MB was significantly degraded in the Fe-WS_2_@GOx+glucose group, suggesting that ROS were generated during the reaction. The formation of gluconic acid led to changes in pH and O_2_ values, which gradually decreased with time in the presence of glucose fixation, confirming the GOx-like activity of Fe-WS_2_@GOx (Figure 2D-E). Given the overexpression of H_2_O_2_ in the bacterial microenvironment, we examined the conversion of H_2_O_2_ to O_2_ using a dissolved oxygen meter to study CAT-like activity (Figure 2F). Compared with the H_2_O group, the Fe-WS_2_@GOx+H_2_O_2_ group was shown significantly higher O_2_ production, suggesting that Fe-WS_2_@GOx triggered the decomposition of H_2_O_2_ to produce O_2_ in the bacterial acidic microenvironment, which was conducive to alleviating bacterial hypoxia. In addition, the GPx-like activity of Fe-WS_2_@GOx was evaluated using a 5,5′-dithiobis-(2-nitrobenzoic acid) (DTNB) assay, which reacted with GSH to form a 5′-thio-2-nitrobenzoic acid (TNB) chromophore with a maximum absorption peak at 412 nm 30. GPx catalyzed the conversion of reduced GSH to GSSG and the subsequent reduction of Fe^3+^ to Fe^2+^ (Figure S4). As shown in Figure 2G, after incubation with Fe-WS_2_@GOx nanozymes, the more GSH was consumed with increasing reaction time and material concentration, confirming that Fe-WS_2_@GOx has excellent GPx-like enzyme activity. Therefore, these results suggest that Fe-WS_2_@GOx nanozymes have multi-enzyme catalytic activity for depleting glucose and GSH while generating H_2_O_2_ within the bacteria, which may lead to bacterial oxidative stress and interruption of nutrient availability.

In order to elucidate the catalytic mechanism of Fe-WS_2_@GOx nanozymes, we further delved into the reaction mechanism and pathways behind their remarkable mimetic enzyme activity using density-functional theory (DFT) calculations. Compared to WS_2_, the W and S XPS spectra of Fe-WS_2_ showed significant negative peak shifts, suggesting that the atoms gained electrons, which increased the electron cloud density and resulted in more excess electrons on the Fe-WS_2_ surface (Figure 2H-I) 31. In addition, the electron localization function (ELF) analysis showed that the free electron gas density of the Fe-WS_2_ structure was significantly lower than that of WS_2_, resulting in more free electron gas (Figure 2J). Electron paramagnetic resonance (EPR) spectroscopy analysis showed a characteristic peak at g = 2.004 for both WS_2_ and Fe-WS_2_, corresponding to the sulfur vacancy signal (Figure 2K). It can be clearly observed that the peak intensity of Fe-WS_2_ was stronger than that of WS_2_, indicating that a strong reaction between Fe and lattice sulfur may have occurred, resulting in more sulfur vacancies. However, the creation of sulfur vacancies leads to changes in the atomic structure, which in turn alters the electronic structure and thus improves the catalytic performance 32. Projection density of states (PDOS) analysis revealed higher electron occupancy near the Fermi energy level in Fe-WS_2_ compared to WS_2_. For the structure of Fe-WS_2_, electrons were easily excited to the conduction band under NIR, proving that the electron transfer in its catalytic process was favorable, potentially facilitating the ROS-catalyzed reaction (Figure 2L). Next, to further elucidate the modulation from these defective sites, we analyzed the contribution of Fe to the WS_2_ band gap through electronic band structure analysis. According to the calculations, the energy level of Fe-WS_2_ (1.0562 eV) was closer to the Fermi energy level than that of WS_2_ (1.4282 eV) due to Fe substitution (Figure 2M).

These results indicated that the substitution of Fe created impurity states within the band gap of WS_2_, which promoted electron generation and reduced the band gap of WS_2_. The reduction of the bandgap facilitates the generation of more photogenerated electrons and accelerates the production of ROS under NIR 33. Subsequently, after identifying the advantages of the defective sites on the surface of the nanozymes, we further explored the potential ROS catalytic mechanisms and activity differences between WS_2_ and Fe-WS_2_. We proposed three potential POD-like catalytic pathways (Figure 2O and S5) and calculated the Gibbs free energies of all the intermediates in each reaction step (Figure 2N). Our results showed that H_2_O_2_ was trapped by WS_2_/Fe-WS_2_ and formed the H_2_O_2_* adsorption state with binding energies of -0.1938 eV for WS_2_ and -0.3023 eV for Fe-WS_2_, indicating that the H_2_O_2_ molecule were readily adsorbed onto Fe-WS_2_, which has many sulfur vacancies. Thus, the superiority of the presence of sulfur vacancies leading to additional defective sites as substrate binding was confirmed in the design of the nanozymes. Subsequently, the spontaneous dissociation of H_2_O_2_* into two *OH radicals at the Fe site with an energy reduction of 0.8727 eV confirmed the thermodynamic feasibility. Afterwards, the generated *OH species can desorb from the surface of the nanozymes and release OH^-^ leading to an increase in energy, which was a rate-determining step. The lower energy barrier for the generation of OH^-^ by Fe-WS_2_ compared to WS_2_ proves that Fe-WS_2_ has a higher catalytic activity for POD-like catalyzation, which was in agreement with the previous experimental results. Thus, DFT calculations suggest that the presence of sulfur vacancies leads to an additional level of native defects produced by the Fe-WS_2_ nanozymes, which facilitates the more formation of electrons on the Fe-WS_2_ surface and promotes ROS generation.

### *In vitro* antibacterial performance of Fe-WS_2_@GOx nanozymes

**Figure 3:**
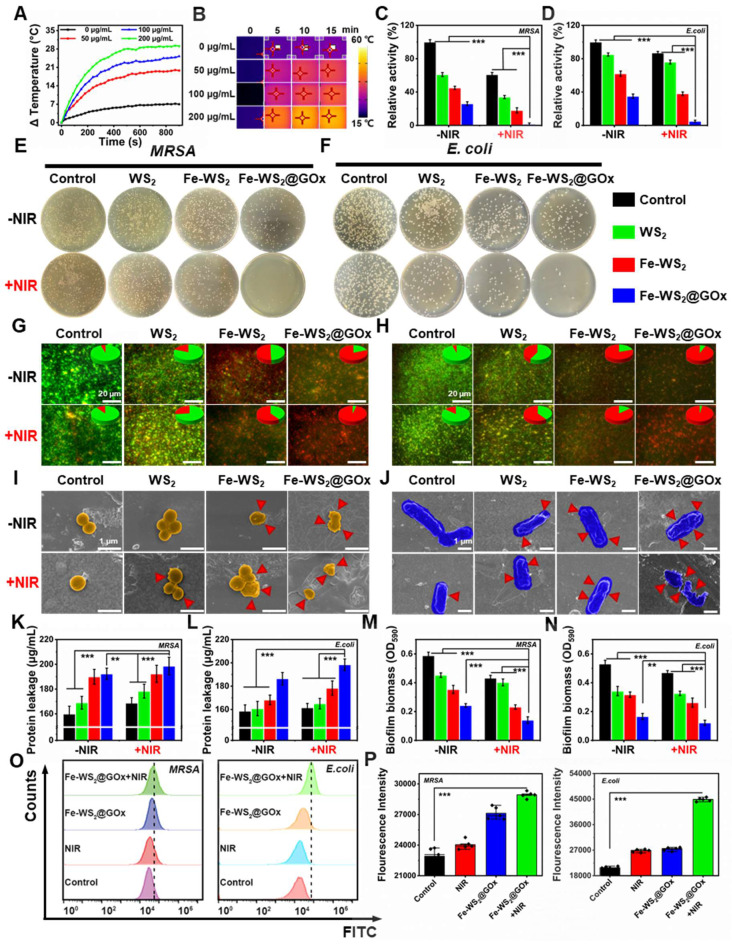
***In vitro* antibacterial performance of Fe-WS_2_@GOx nanozymes.** (A) Temperature profiles of different concentrations of Fe-WS_2_ at 808 nm NIR and (B) corresponding NIR images. (C-F) Pictures of colonies of MRSA and *E. coli* agar plates after different treatments and statistics of the number of surviving bacteria. (G-H) Live/dead staining of MRSA and *E. coli* after different treatments. Scale bar = 20 μm. SEM images of (I) MRSA (Scale bar = 1 μm) and (J) *E. coli* (Scale bar = 1 μm) (red arrows indicate ruptured bacteria). (K-L) Protein leakage and (M-N) aqueous crystalline violet staining of MRSA and *E. coli* after different treatments. (O) Flow cytometry detection of MRSA and* E. coli* membrane potential changes and (P) semi-quantitative analysis of fluorescence intensity. N = 3. (P values based on Student's test: *P ≤ 0.05, **P ≤ 0.01, ***P ≤ 0.001).

It is reported that NIR can increase the electron transfer rate 34. Therefore, we next explored the promoted electron transfer efficiency of defectively engineered Fe-WS_2_ nanozymes with strong NIR absorbance at the bacterial level. First, we explored whether Fe-WS_2_ nanozymes could synergize with NIR to promote ROS production. Under NIR irradiation, the Fe-WS_2_ with abundant sulfur vacancies can generate more ROS by altering the H_2_O_2_ concentration or altering the laser power (Figure S6). This phenomenon may be attributed that near-infrared radiation can effectively promote their electron transfer process 35. Subsequently, we investigated the Fe-WS_2_ photothermal conversion ability. By varying the concentration of Fe-WS_2_, the temperature of the control group only increased by about 6 °C, whereas the temperature of the 200 μg/mL Fe-WS_2_ group increased by nearly 29 °C. These phenomena were further confirmed by the corresponding IR thermal images of the different groups (Figure 3A-B). In addition, the temperature of the Fe-WS_2_ nanozymes aqueous solution increased gradually with increasing 808 nm laser power density (Figure S7). This indicated that Fe-WS_2_ has excellent photothermal conversion performance. The negligible changes in the photothermal profiles over five heating and natural cooling cycles indicate that Fe-WS_2_ has excellent photothermal stability (Figure S8). In summary, Fe-WS_2_ nanozymes exhibit significant photothermal conversion performance under NIR irradiation for enhanced enzyme-like catalytic activity.

Due to the excellent photothermal conversion performance of Fe-WS_2_@GOx nanozymes, we further explored their *in vitro* antibacterial effects. First, we explored the bactericidal effect of Fe-WS_2_@GOx nanozymes against MRSA and *E. coli* by plate counting method. We categorized the bacterial solutions into eight groups: PBS, NIR, WS_2_, WS_2_+NIR, Fe-WS_2_, Fe-WS_2_+NIR, Fe-WS_2_@GOx and Fe-WS_2_@GOx+NIR eight groups. Figure 3C-F showed the antimicrobial effect of different treatments against MRSA and *E. coli*. Notably, the Fe-WS_2_@GOx+NIR group significantly inhibited bacterial growth, demonstrating that Fe-WS_2_@GOx has excellent enzyme-like activity and produces a large amount of ROS, leading to bacterial oxidative stress. The results of live/dead staining assay (Figure 3G-H) further confirmed the excellent antibacterial effect of Fe-WS_2_@GOx+NIR. The highest bacterial mortality rate was observed in the Fe-WS_2_@GOx+NIR group, and there were almost no live bacteria, indicating that all the bacteria were killed. In addition, scanning electron microscopy (SEM) was used to observe the morphological changes after different treatments. Under NIR irradiation, both Fe-WS_2_ and Fe-WS_2_@GOx groups severely damaged MRSA and *E. coli*, with the Fe-WS_2_@GOx+NIR group causing extensive bacterial membrane rupture, demonstrating complete bacterial destruction (Figure 3I-J). Subsequently, the disruption of the cell membrane was further verified by monitoring the leakage of proteins (Figure 3K-L). The Fe-WS_2_@GOx+NIR group showed the highest amount of protein detected, confirming that Fe-WS_2_@GOx+NIR caused severe disruption to the bacterial cell membrane. However, biofilm clearance was more challenging than eliminating free-state bacteria due to the protective effect of extracellular polymeric substances (EPS), which hindered antimicrobial drug penetration 36. Therefore, we used crystal violet staining to quantitatively determine the extent of biofilm destruction (Figure S9). As shown in Figure 3M-N, Fe-WS_2_@GOx+NIR had the most pronounced inhibitory effect on biofilm formation induced by MRSA and *E. coli*. Subsequently, Membrane potential changes in MRSA and *E. coli* were detected by flow cytometry (Figure 3O-P). The Fe-WS_2_@GOx+NIR group exhibited stronger fluorescence intensity compared to the control, NIR and Fe-WS_2_@GOx groups. These results demonstrate that defect-engineered Fe-WS_2_@GOx exhibits excellent antibacterial activity under NIR irradiation, effectively eradicating both planktonic bacteria and biofilms.

### *In vitro* mechanistic study of Fe-WS_2_@GOx nanozymes

**Figure 4:**
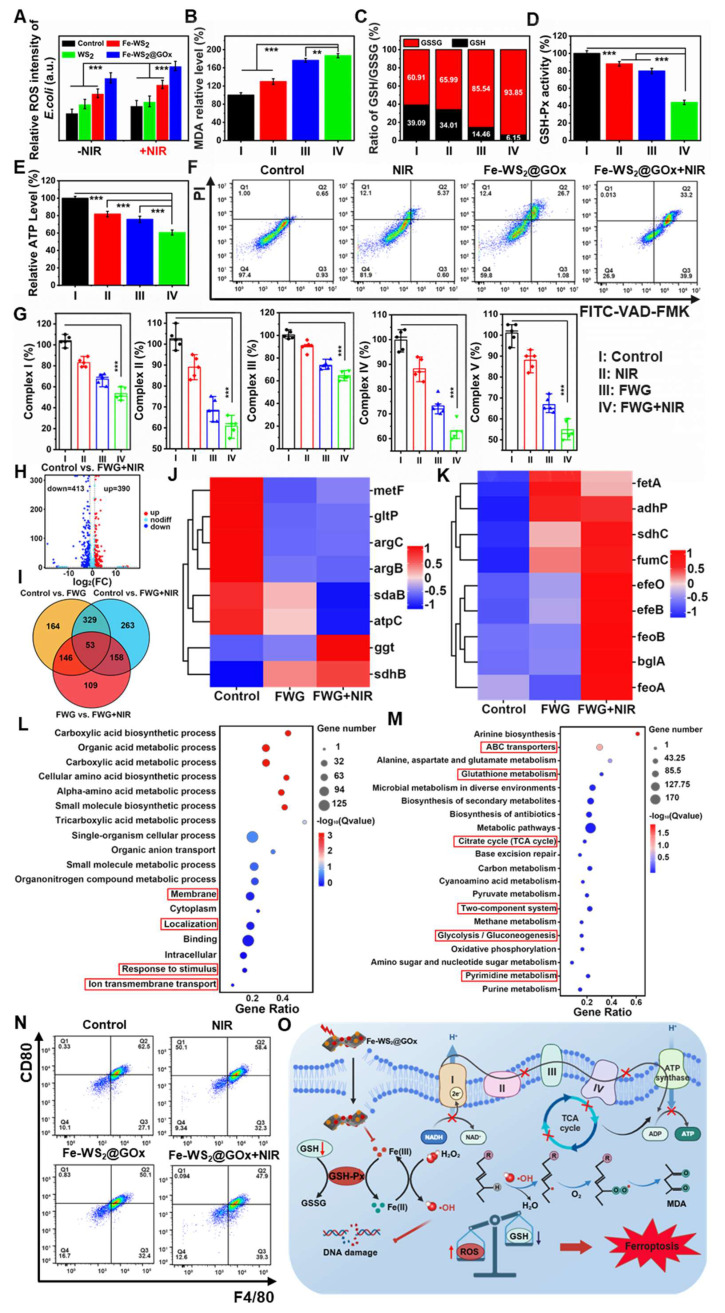
***In vitro* mechanistic studies of Fe-WS_2_@GOx nanozymes.** (A) Detection of ROS content in *E. coli* after different treatments using DCFH-DA probe. (B) MDA and (C) GSH/GSSG level in *E. coli* after different treatments. (D) GSH-Px activity. (E) ATP content in *E. coli*. (F) Apoptosis of *E. coli* after different treatments analyzed by flow cytometry. (G) Effect of different treatments on the activity of respiratory chain complexes. (H) Volcano plot of the distribution of DEGs in Control compared with Fe-WS_2_@GOx+NIR. (I) Summarization of target genes in different groups. (J) Heat map of metabolism-related genes. (K) Heat map of iron transport-related genes. (L) GO analysis of DEGs. (M) KEGG analysis of DEGs. (N) Flow cytometry analysis of CD80 expression in different treated macrophages. (O) Schematic diagram of the antimicrobial mechanism. (I) Control, (II) NIR, (III) Fe-WS_2_@GOx, and (IV) Fe-WS_2_@GOx+NIR. N = 3. (P values based on Student's test: *P ≤ 0.05, **P ≤ 0.01, ***P ≤ 0.001).

After determining that Fe-WS_2_@GOx has an antimicrobial effect, *in vitro* experiments were conducted to further investigate the mechanism. 2′,7′-dichlorodihydrofluorescein diacetate (DCFH-DA) assay was applied to detect intracellular ROS formation in different groups. After different treatments of *E. coli* and MRSA, we found that the Fe-WS_2_@GOx+NIR group induced the greatest fluorescence intensity of ROS, which further proved that Fe-WS_2_@GOx with the synergistic effect of NIR had a strong antibacterial effect by regulating the catalytic activity and promoting the production of ROS (Figure 4A and S10). Ferroptosis is a new type of iron-dependent programmed death accompanied by lipid peroxidation 37. The lipid peroxidation level was monitored using the MDA detection kit experiment, confirming the lipid peroxidation process induced by the Fe-WS_2_@GOx+NIR (Figure 4B and S11A). GSH levels are considered biochemical markers for evaluating the degree of ferroptosis and are negatively correlated with ferroptosis 38. The GSSG/GSH ratio in *E. coli* and MRSA was measured across treatment conditions. The Fe-WS_2_@GOx+NIR group showed the highest GSSG/GSH ratio along with significantly decreased GSH-Px activity (Figure 4D and S12A), indicating GSH depletion and conversion to GSSG (Figure 4C and S11B). The above results demonstrate that under NIR irradiation, Fe-WS_2_@GOx generates a substantial amount of ROS, which react with GSH within bacteria, inactivating GSH-Px and leading to the accumulation of lipid peroxides, thereby inducing ferroptosis in bacteria. At the same time, the respiratory chain of the bacteria was also affected by the ferroptosis. ATP, as the most important energy currency for bacteria, its synthesis inhibition will seriously interfere with the ETC of bacteria 39. To investigate the mechanism by which Fe-WS_2_@GOx interferes with the bacterial ETC under NIR irradiation, we examined its effects on key respiratory chain enzymes and ATP synthesis. Firstly, the ATP content was detected by using the kit after different treatments, revealing the most significant reduction in the Fe-WS_2_@GOx+NIR group (Figure 4E and S12B). Subsequently, we explored whether the bacterial ETC was disrupted by detecting the activity of five key enzymes in the bacterial respiratory chain complex (NADH-fumarate oxidoreductase, succinate-fumarate oxidoreductase, fumarate-cytochrome c oxidoreductase, cytochrome c oxidase, and ATP synthase) (Figure 4G). Meanwhile, the apoptosis of *E. coli* was analyzed by flow cytometry using FITC-VAD-FMK and PI staining, and the apoptosis rate was significantly higher in the Fe-WS_2_@GOx+NIR group (Figure 4F). In summary, under NIR irradiation, Fe-WS_2_@GOx induces bacterial ferroptosis by generating ROS while inhibiting bacterial ETC function, ultimately leading to bacterial apoptosis.

To further explore the “electron transport chain interference” amplified bacterial ferroptosis mechanism of Fe-WS_2_@GOx against *E. coli*, the whole transcriptome RNA sequencing was assessed. Preliminary identification of differentially expressed genes (DEGs) between the control group and the Fe-WS_2_@GOx+NIR group was performed. The volcano plot showed that the Fe-WS_2_@GOx+NIR group had 803 DEGs compared to the control group (Figure 4H). Meanwhile, there were also gene expression differences in control vs. Fe-WS_2_@GOx and control vs. Fe-WS_2_@GOx+NIR (Figure 4I and S13). The SDHB gene encodes the iron-sulfur protein subunit of succinate dehydrogenase, which is a key component of the mitochondrial respiratory chain complex II (SDH) and also plays an important role in the tricarboxylic acid (TCA) cycle 40. The expression heat map showed that sdhB expression was upregulated, indicating that Fe-WS_2_@GOx+NIR can generate a large amount of ROS, which can effectively disrupt the ETC of *E. coli*, leading to severe inhibition of bacterial energy metabolism (Figure 4J). Iron ions accumulate in bacteria due to enhanced uptake and limited efflux, thereby activating a series of signaling pathways that promote bacterial ferroptosis. The heatmap revealed significant upregulation of transferrin-related genes, suggesting enhanced bacterial iron uptake promotes ferroptosis induction (Figure 4K). Subsequently, we used Gene Ontology (GO) and Kyoto Encyclopedia of Genomes (KEGG) analyses to investigate possible pathways. GO enrichment analysis revealed that DEGs in the *E. coli* group were involved in biological processes, cellular components, and molecular functions, suggesting that Fe-WS_2_@GOx+NIR induced the stress response (Figure S14). GO analysis further revealed altered expression of bacterial metabolic genes (Figure 4L). Meanwhile, the expression of TCA cycle, glutathione metabolism and glycolysis/gluconeogenesis genes were also significantly changed by KEGG analysis (Figure 4M). Based on the above findings, Fe-WS_2_@GOx+NIR can generate ROS by inducing bacterial ferroptosis and disrupting the bacterial ETC. These induced effects alter bacterial membrane permeability, leading to increased Fe^2+^ uptake by bacteria, triggering oxidative stress, and impairing energy metabolic processes (Figure 4O).

In addition to the antimicrobial mechanism, the role of Fe-WS_2_@GOx as an immunomodulator was also explored. First, the biosafety of Fe-WS_2_@GOx was evaluated. L929 cells (mouse fibroblasts) were used as a normal cell control to investigate the cytotoxicity of Fe-WS_2_@GOx (Figure S15). It was shown by CCK-8 assay that Fe-WS_2_ and Fe-WS_2_@GOx were less cytotoxic to normal cells under low-concentration conditions. In addition, the biosafety was further verified by hemolysis assay (Figure S16). The results demonstrated that Fe-WS_2_@GOx exhibited favorable biocompatibility, suggesting its potential applicability in wound treatment. During tissue repair, the polarization of macrophages from M1 to M2 phenotype could promptly suppress the inflammatory response at the wound and accelerate the wound repair process. Subsequently, RAW264.7 was stimulated with LPS (lipopolysaccharide) to form the M1 phenotype, which was then examined by flow cytometry after different treatments (Figure 4N and S17). The Fe-WS_2_@GOx+NIR group showed significantly lower CD80 expression but higher CD206 expression compared to other groups, indicating its ability to promote M2 phenotype of RAW264.7 cells. These results indicate that Fe-WS_2_@GOx exhibits high biocompatibility and induces the transformation of the M1 phenotype to the M2 phenotype, thereby promoting angiogenesis and tissue regeneration to potentially accelerate wound healing.

### *In vivo* wound healing properties of Fe-WS_2_@GOx

**Figure 5:**
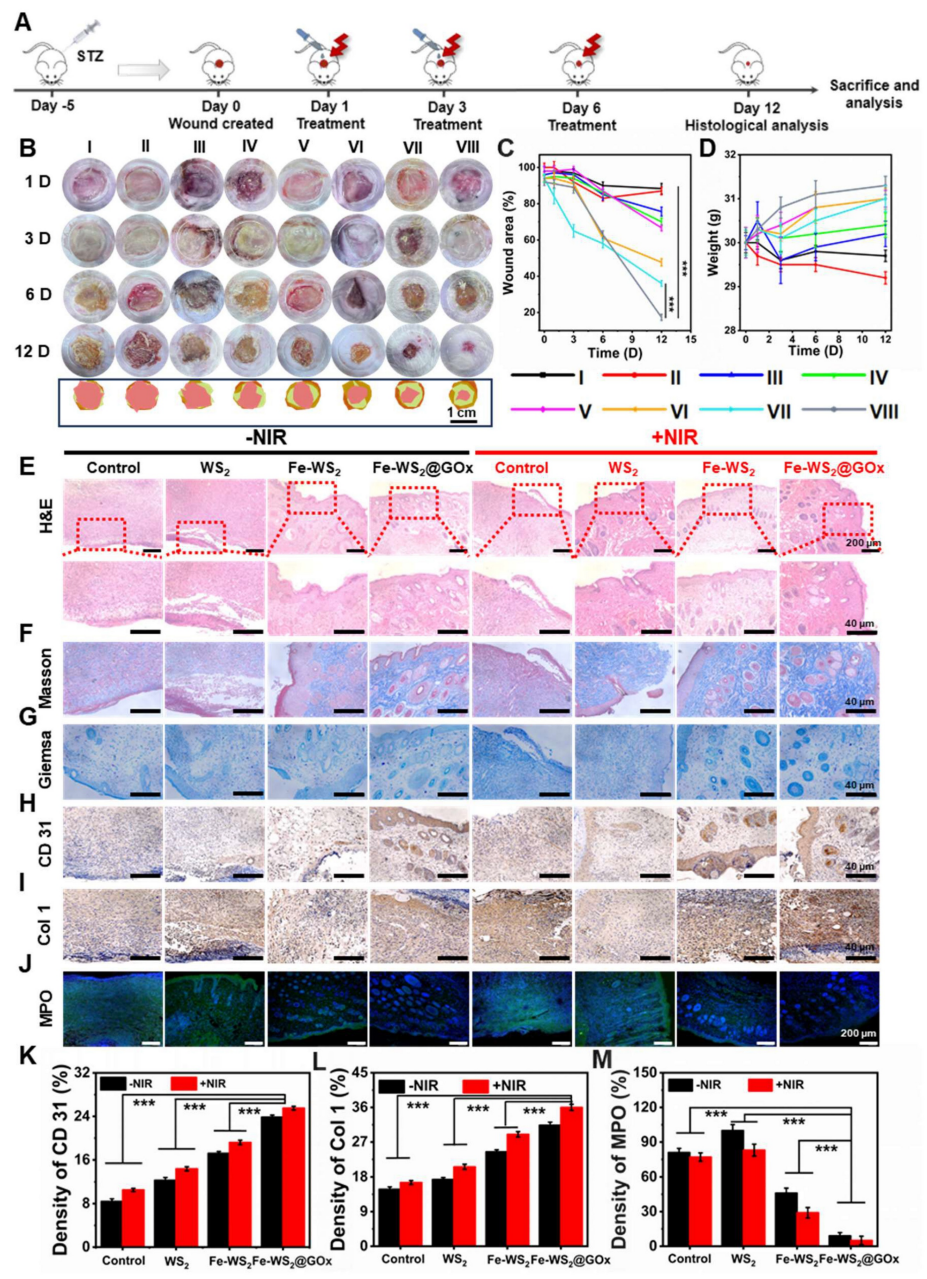
** Efficacy of Fe-WS_2_@GOx in a diabetic wound infection model.** (A) Schematic of the treatment strategy in the MRSA-infected diabetic mouse wound model. (B) Wound images and (C) quantitative analysis of wound area from day 1 to day 12 after different treatments. (D) Changes in body weight of mice. (E-G) H&E, Masson and Giemsa staining of wound tissue. (H-I) Immunohistochemical staining and (K-L) semi-quantitative analysis of CD31 and Col 1 in wound tissue. Scale bar = 40 μm. (J) Immunofluorescence staining and (M) semi-quantitative analysis of MPO in wound tissue. Scale bar = 200 μm (I) Control, (II) NIR, (III) WS_2_, (IV) WS_2_+NIR, (V) Fe-WS_2_, (VI) Fe-WS_2_+NIR, (VII) Fe-WS_2_@GOx, and (VIII) Fe-WS_2_@GOx+NIR. N = 3. (P values based on Student's test: *P ≤ 0.05, **P ≤ 0.01, ***P ≤ 0.001).

Inspired by the excellent antimicrobial efficacy and immunomodulatory effects *in vitro*, we further constructed a diabetic mouse model of bacterial infection to evaluate the *in vivo* diabetic wound healing properties of Fe-WS_2_@GOx. The comprehensive process of *in vivo* treatment was shown in Figure 5A. Rats were randomly assigned to eight groups with different treatment groups: (I) Control; (II) WS_2_; (III) Fe-WS_2_; (IV) Fe-WS_2_@GOx; (V) NIR; (VI) WS_2_+NIR; (VII) Fe-WS_2_+NIR; (VIII) Fe-WS_2_@GOx+NIR. 5 μL of different materials (50 μg/mL) were dropped into the infection site of each group of mice. Then, the mouse wounds were irradiated with a near-infrared light source at a power density of 1 W/cm^2^ for 10 minutes, and temperature changes were monitored using an infrared thermography camera (Figure S18). Figure 5B-C was demonstrated the wound healing of different treatment groups over time. Impressively, the Fe-WS_2_@GOx+NIR group showed the fastest wound healing, which could be attributed to the superior antimicrobial effect and immunomodulatory effect of Fe-WS_2_@GOx+NIR. In addition, its biocompatibility was verified by monitoring body weight (Figure 5D), H&E staining of major organs and routine blood tests (Figure S19 and S20). Subsequently, to further evaluate the therapeutic effects, hematoxylin and eosin (H&E), Masson, Giemsa, immunohistochemistry, and immunofluorescence staining were performed on the dorsal wound tissues of the mice after 12 days of treatment. As observed by H&E staining, the Fe-WS_2_@GOx+NIR group recovered well without significant inflammatory reaction (Figure 5E). Masson staining was used to assess the aggregation of collagen fibers in regenerated skin tissues (Figure 5F). The collagen in the Fe-WS_2_@GOx+NIR group was the most colorful and densely packed in the structure, indicating that Fe-WS_2_@GOx+NIR accelerated the collagen deposition and remodeling. In addition, collagen production was further verified by immunohistochemical examination of Col 1 (Figure 5I and L). It was observed that the Fe-WS_2_@GOx+NIR group exhibited stronger Col 1 signals compared to the other groups, further confirming its ability to promote tissue repair. Next, it was confirmed by Giemsa staining that the Fe-WS_2_@GOx+NIR group significantly eliminated bacterial invasion of the tissue after day 12 (Figure 5G). In addition, CD31, a transmembrane protein expressed in early angiogenesis, can be used to assess newly formed blood vessels in wound healing 41. Immunohistochemical staining showed that CD31 was less expressed in the control group and most strongly expressed in the Fe-WS_2_@GOx+NIR group (Figure 5H and K). It has been reported that the formation of NET can lead to the persistence of biofilm infections, and the low expression of MPO has an inhibitory effect on NET 42. By immunofluorescence detection, MPO expression was low in all experimental groups, but MPO expression was lowest in the Fe-WS_2_@GOx group and the Fe-WS_2_@GOx+NIR group, suggesting that Fe-WS_2_@GOx had an inhibitory effect on NET and accelerated wound healing (Figure 5J and M). Taken together, these results clearly demonstrated that Fe-WS_2_@GOx could accelerate the healing ability of diabetic infected wounds by efficiently removing MSRA and promoting angiogenesis *in vivo*.

**Figure 6:**
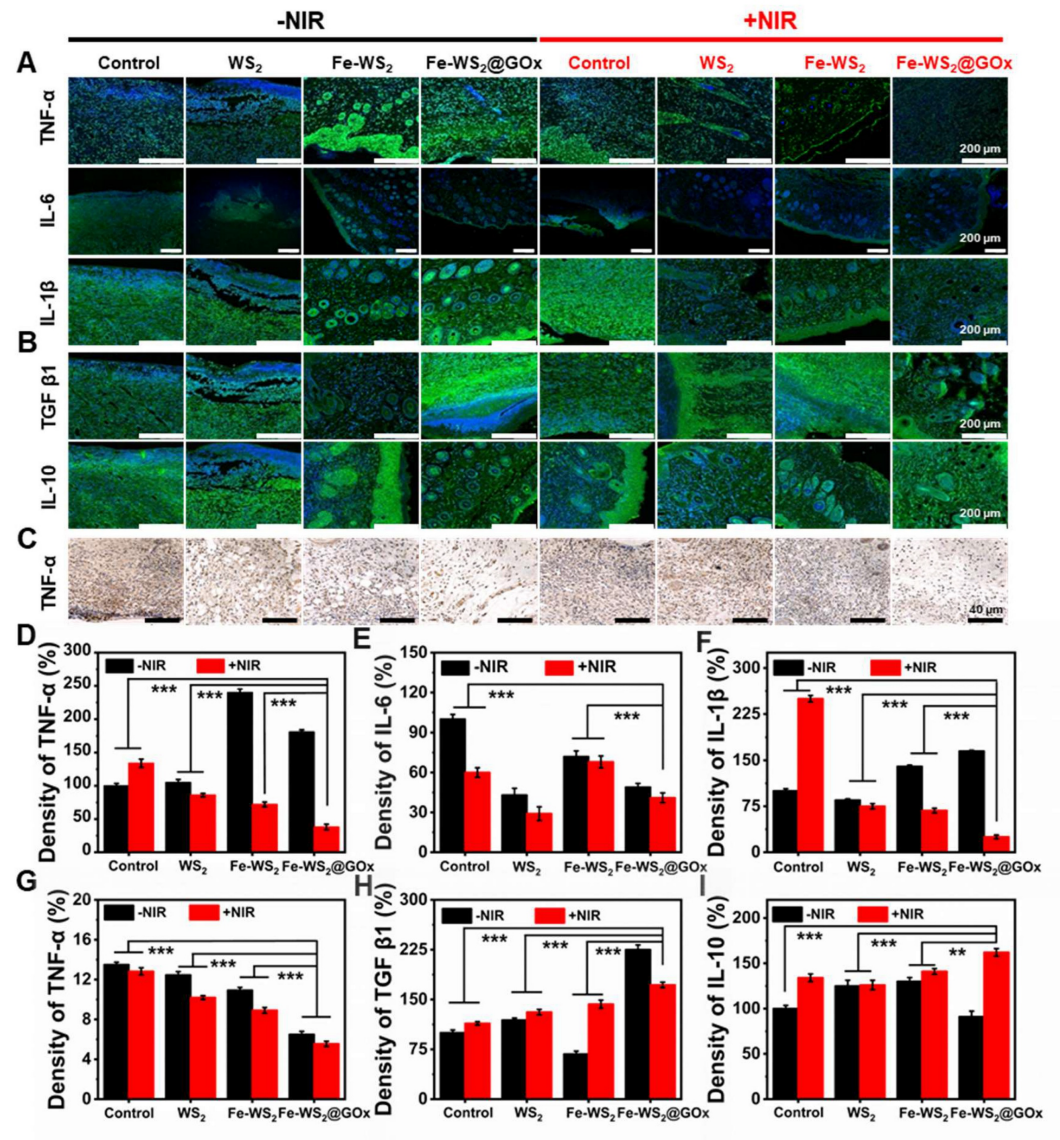
** Assessment of inflammatory factors on day 12.** (A) Immunofluorescence staining and (D-F) semi-quantitative analysis of pro-inflammatory cytokines (TNF-α, IL-6 and IL-1β). Scale bar = 200 μm. (B) Immunofluorescence staining and (H-I) semi-quantitative analysis of anti-inflammatory cytokines (TGF β1 and IL-10). Scale bar = 200 μm. (C) Immunohistochemical staining for TNF-α secretion and (G) semi-quantitative analysis. Scale bar = 40 μm. N = 3. (P values based on Student's test: *P ≤ 0.05, **P ≤ 0.01, ***P ≤ 0.001).

A key cause of impaired diabetic wound healing is a sustained inflammatory response due to excess inflammatory factors 43. Subsequently, we evaluated the expression of pro-inflammatory and anti-inflammatory factors in wound tissues using immunofluorescence and immunohistochemistry staining to assess the inflammatory status and investigate the intrinsic mechanisms underlying wound healing. The staining results were shown in Figure 6A and C. The high expression levels of tumor necrosis factor α (TNF-α), interleukin 6 (IL-6), and interleukin 1β (IL-1β) in the wound tissues of control diabetic mice were attributed to the severe inflammatory response. Compared with the control group, the expression of TNF-α, IL-6 and IL-1β was significantly lower in the Fe-WS_2_@GOx+NIR group, which was mainly attributed to the inhibitory effect of Fe-WS_2_@GOx+NIR on the inflammatory response. Also, the quantitative statistics of fluorescence intensity confirmed this result (Figure 6D-G). In contrast, the expression of anti-inflammatory cytokines, including transforming growth factor β1 (TGF β1) and interleukin 10 (IL-10) was markedly elevated in the Fe-WS_2_@GOx+NIR group relative to the control group (Figure 6B). This suggested that Fe-WS_2_@GOx+NIR has excellent antibacterial and anti-inflammatory effects, which can alleviate the inflammatory response at the wound site and establish an anti-inflammatory microenvironment (Figure 6H-I). In conclusion, Fe-WS_2_@GOx with NIR irradiation can effectively alleviate the inflammatory condition and accelerate wound healing in the wound.

**Figure 7:**
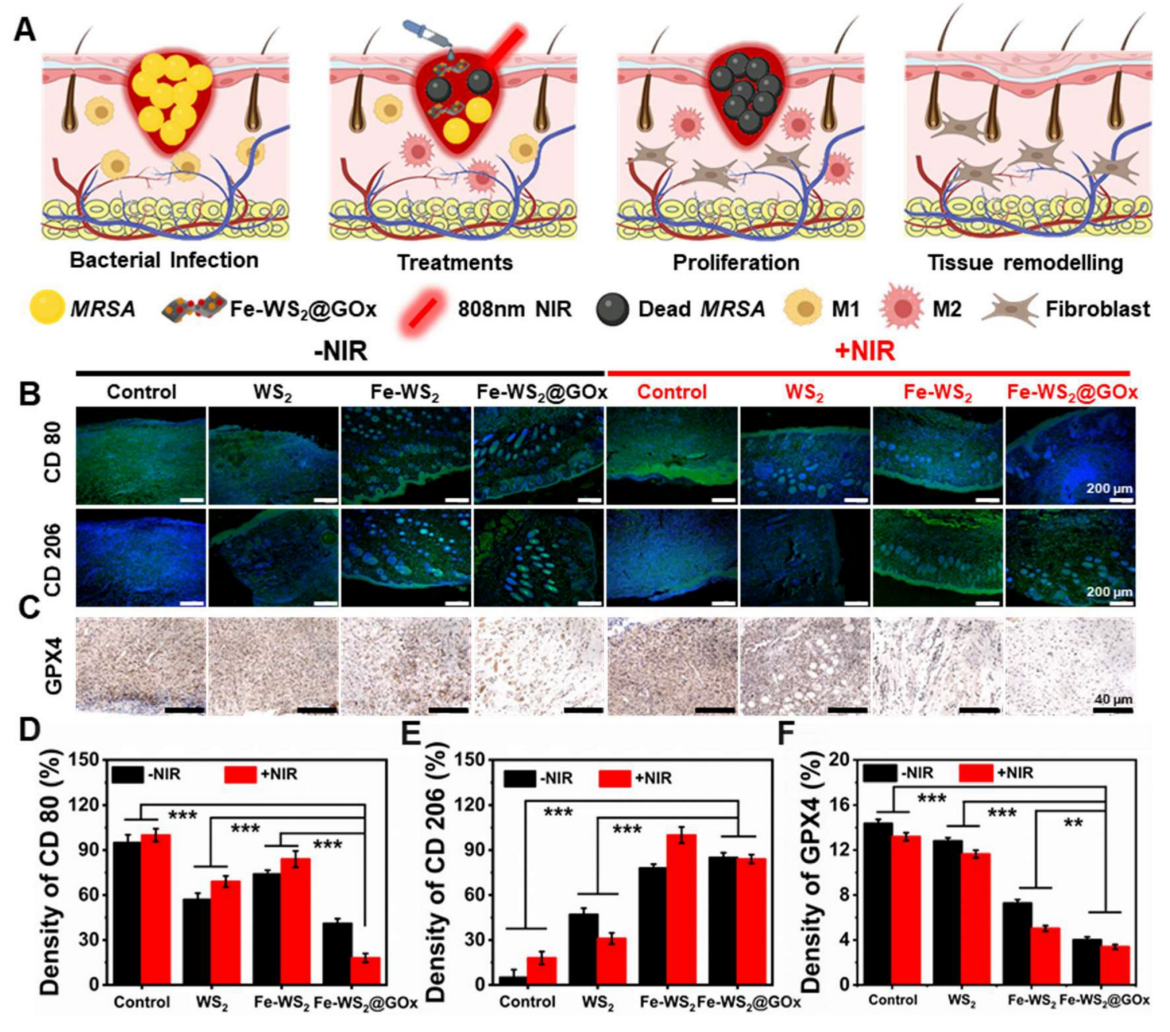
** Fe-WS_2_@GOx mediates intrinsic drivers of wound healing.** (A) Illustration of the healing process of diabetic infected wounds in mice. (B) Immunofluorescence staining and (D-E) semi-quantitative analysis of CD80 and CD206 after different treatments. Scale bar = 200 μm. (C) Immunofluorescence staining and (F) semi-quantitative analysis of ferroptosis marker GPX4 after different treatments. Scale bar = 40 μm. N = 3. (P values based on Student's test: *P ≤ 0.05, **P ≤ 0.01, ***P ≤ 0.001).

To make this more convincing, we next evaluated the *in vivo* immunomodulatory effects of Fe-WS_2_@GOx nanozymes. The schematic diagram in Figure 7A illustrated the application of Fe-WS_2_@GOx+NIR therapeutic platform in diabetic wound healing, which usually proceeds in a chronological order, namely bacterial infection, treatments, proliferation, and tissue remodeling. Timely switching of macrophages from the M1 to the M2 phenotype plays a vital role in tissue repair and regeneration. Detection of CD80 and CD206 by immunofluorescence staining revealed that the expression of CD80 was almost absent in the Fe-WS_2_@GOx+NIR group, whereas the expression of CD206 was significantly increased compared with the control group (Figure 7B). Meanwhile, semi-quantitative analysis led to the same conclusion (Figure 7D-E). This implied that the nanozymes promoted macrophage polarization to the M2 phenotype, which played a functional role in the suppression of inflammatory responses and immunomodulation. GPX4 is a GSH-dependent antioxidant, and its inactivation is a key contributor to the onset of ferroptosis 44, 45. Further, the expression level of GPX4 was examined by *in vivo* immunohistochemistry. The results showed that Fe-WS_2_@GOx nanozymes decreased GPX4 expression, and this down-regulation was enhanced by co-NIR irradiation (Figure 7C and F), emphasizing the critical role of ferroptosis in Fe-WS_2_@GOx-induced bacterial death.

## Conclusion

The diabetic microenvironment leads to persistent wound infections that can impede wound healing. This synthesized Fe-WS_2_@GOx nanozymes with defect engineering can simultaneously alleviate hyperglycemic and hypoxic microenvironment by continuously endogenous glucose consumption, H_2_O_2_ cyclic accumulation and O_2_ sustainable supplement, thereby interfering with the bacterial ETC and inducing ferroptosis, ultimately promoting the healing of diabetic wounds. Additionally, the generated ROS disrupt the ETC of bacteria by inhibiting the activity of ETC complexes and blocking electron transfer. Notably, Fe-WS_2_@GOx nanozymes can promote angiogenesis and tissue regeneration and accelerate diabetic wounds healing through inflammatory factor expression and immunomodulation of macrophage polarization. In conclusion, this study opens new avenues for the treatment of diabetic wound infection pathology by designing nanozymes that coordinate ETC interference and induce ferroptosis as well as wound tissue repair.

## Supplementary Material

Supplementary figures.
