# Supplementary material for: “Electron transport chain interference” strategy of amplified bacterial ferroptosis and defect-engineered nanozyme for diabetic wound healing
Source: Theranostics. 2026 Jan 1;16(3):1262–80. doi: 10.7150/thno.121636 (PMC12679372; doi:10.7150/thno.121636)
Supplement: Supplementary file 1 — Supplementary figures. [file thnov16p1262s1.pdf]

Supporting information

"Electron transport chain interference" strategy of amplified bacterial ferroptosis and defect-engineered nanozyme for diabetic wound healing

Yanlan Xie[1#], Huan Wang[1#], Yalan Wang[1], Jiajie Liu[1], Jinming Tong[2], Tao Wu[2], Li Yin[2], Chuan Zhang[2,3*], Long Zhao[2,4*], Yuan Yong[1,2*]

1 Key Laboratory of Pollution Control Chemistry and Environmental Functional Materials for Qinghai-Tibet Plateau of the National Ethnic Affairs Commission, School of Chemistry and Environment, Southwest Minzu University, Chengdu 610041, China

2 Nanomedicine Innovation Research and Transformation Institute, Affiliated Hospital of North Sichuan Medical College, Nanchong 637000, China

3 Biotechnology Innovation Drug Application and Transformation Key Laboratory of Sichuan Province, North Sichuan Medical College, Nanchong 637000, China

4 Department of Neurosurgery, Affiliated Hospital of North Sichuan Medical College, Nanchong 637000, China

# Yanlan Xie and Huan Wang contributed equally to the work.

Corresponding Authors: yongy1816@163.com; cbyzhaolong@163.com; zhangchuanforever@yeah.net

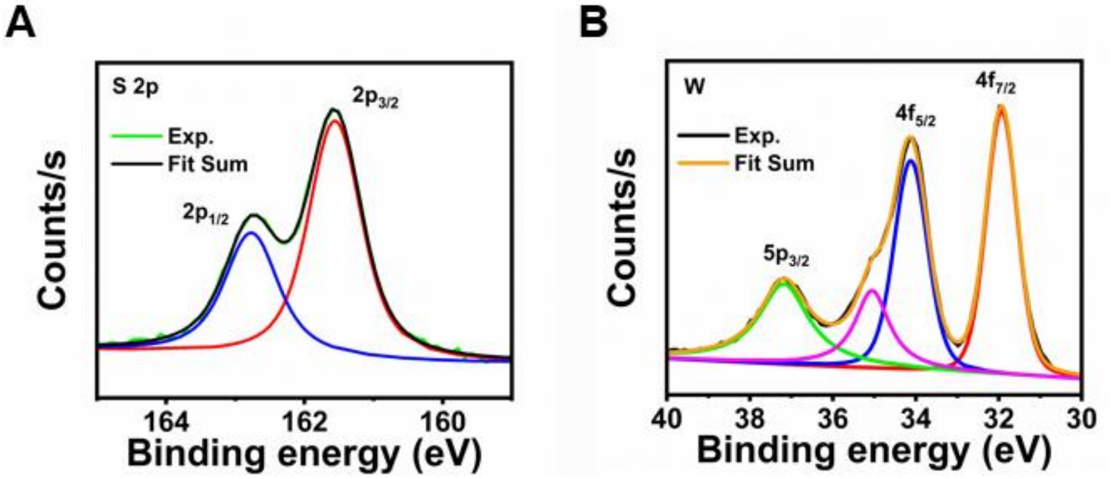

Figure S1. XPS fine spectrum of elements W and S in $Fe-WS_2$.

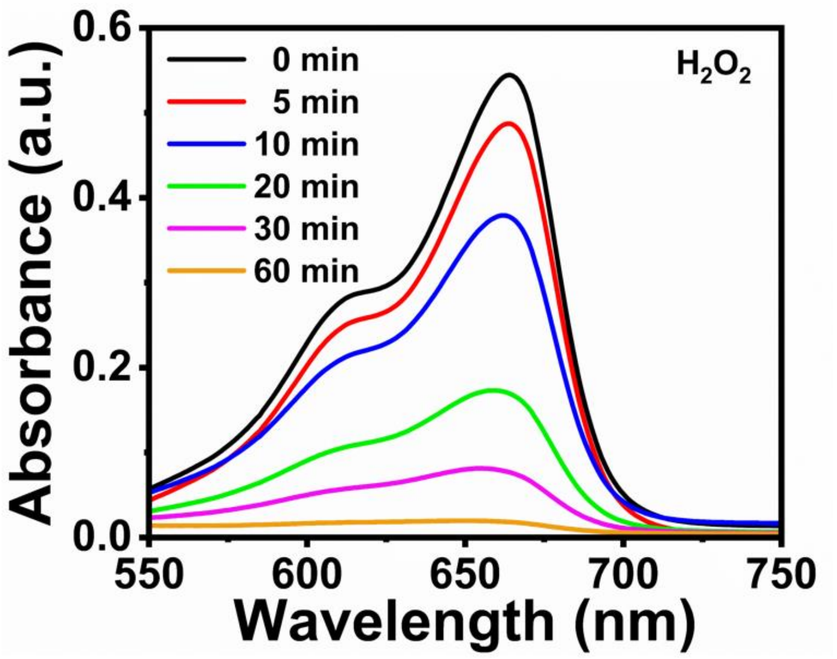

Figure S2. The degradation of MB absorbance was measured at different reaction times.

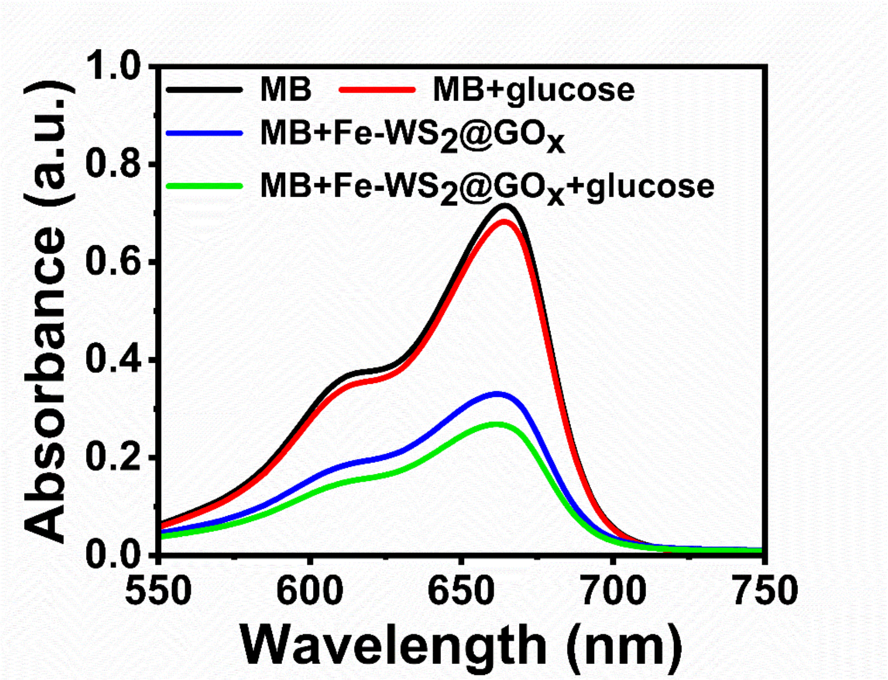

Figure S3. GOx-like enzyme activity of Fe-WS_2@GOx using MB chromogen.

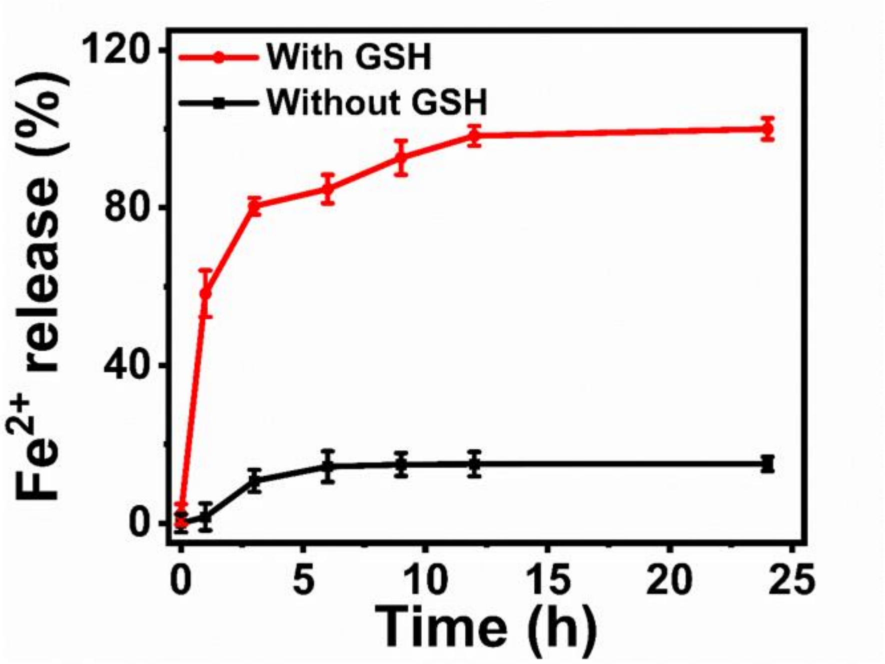

Figure S4. Fe^{2+} release was determined over time in the presence/absence of GSH.

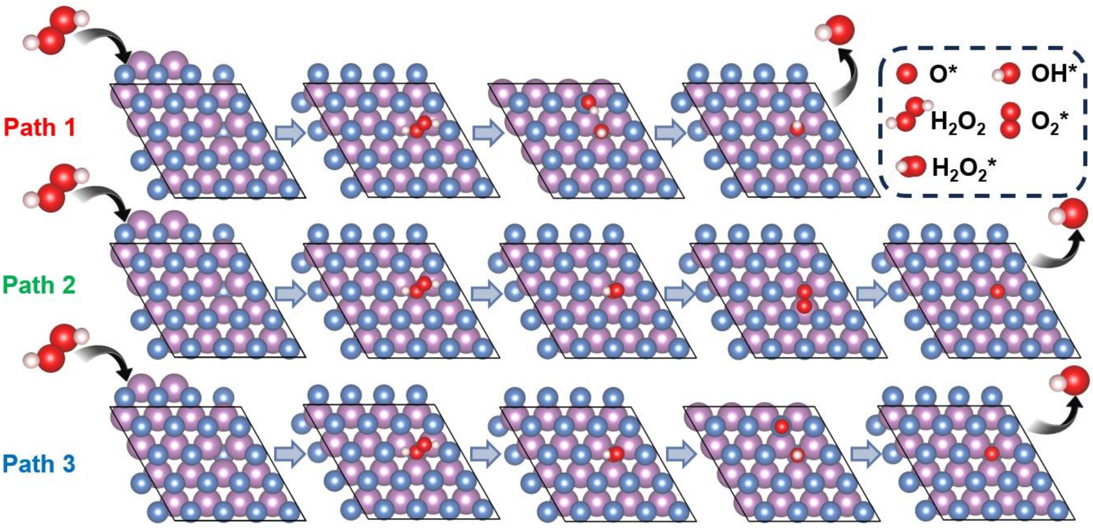

Figure S5. The corresponding reaction pathway for H_2O_2-catalyzed WS_2.

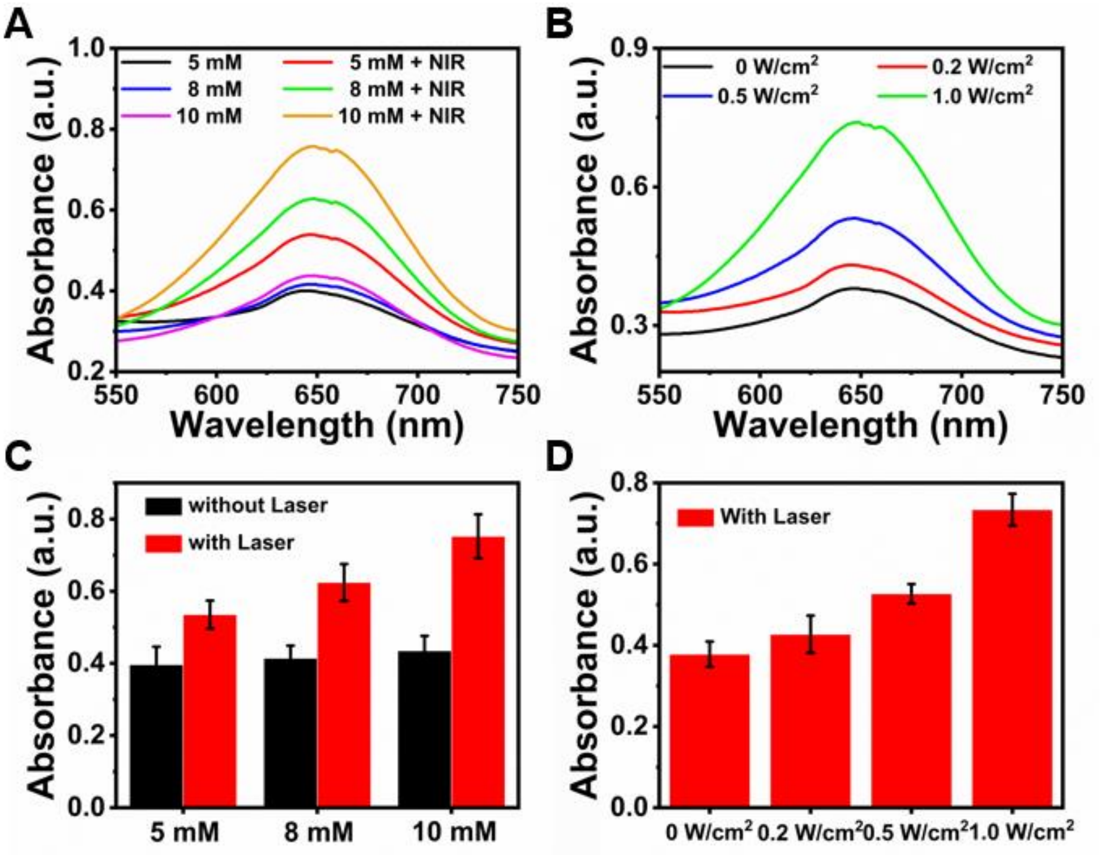

Figure S6. (A, C) UV absorption of Fe-WS_2 by varying H_2O_2 concentration in the presence or absence of 808 nm NIR irradiation. (B, D) UV absorption of Fe-WS_2 by varying laser power density in the presence or absence of 808 nm NIR irradiation.

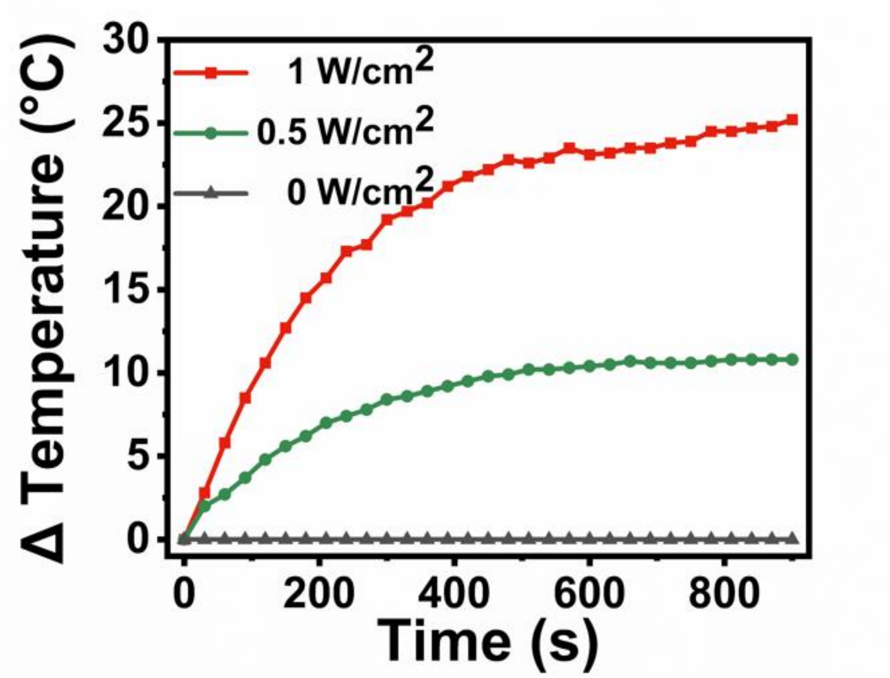

Figure S7. Photothermal effects of Fe-WS_2 nanozymes at different laser powers.

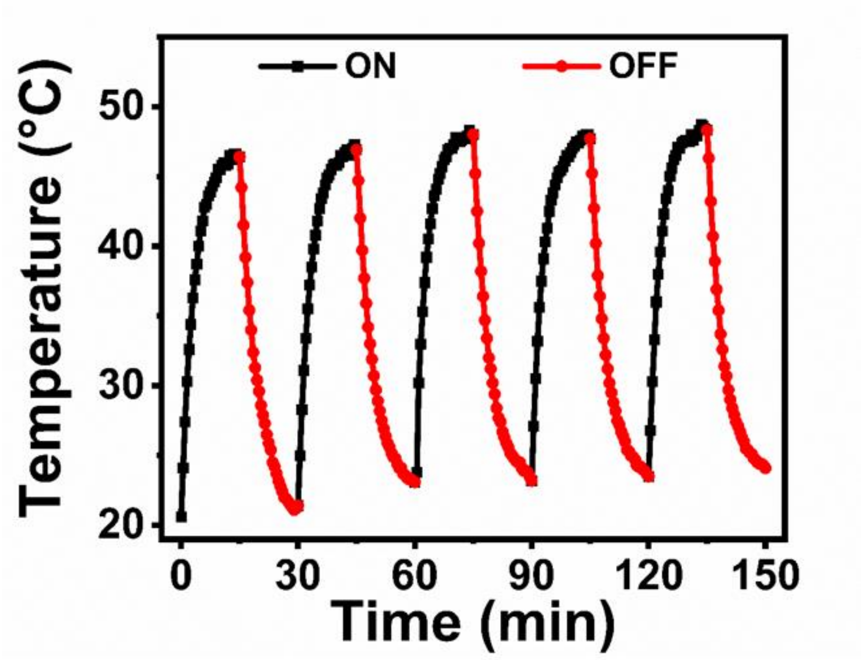

Figure S8. Five photothermal cycles of Fe-WS_2 at 100 μg/mL in the NIR (808 nm, 1 W/cm^2).

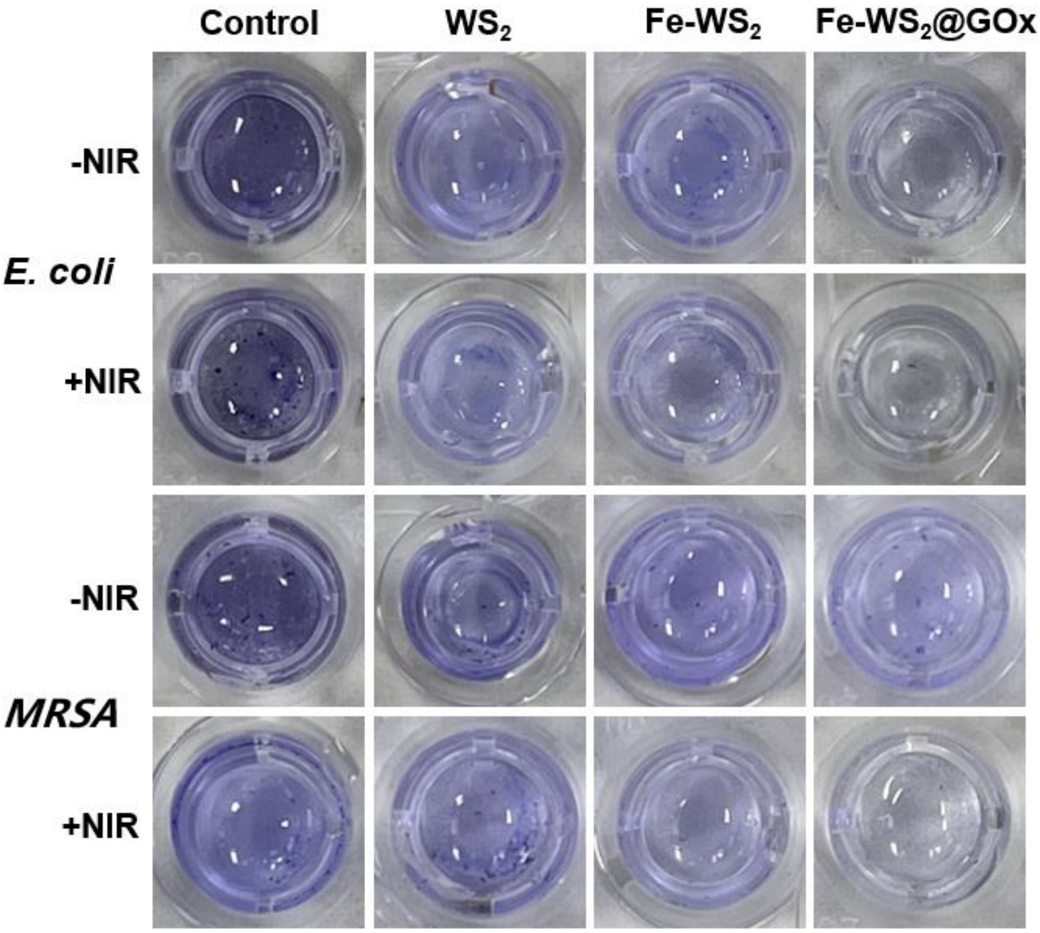

Figure S9. Bacterial biofilms stained with crystal violet dye after different treatments of *E. Coli* and MRSA.

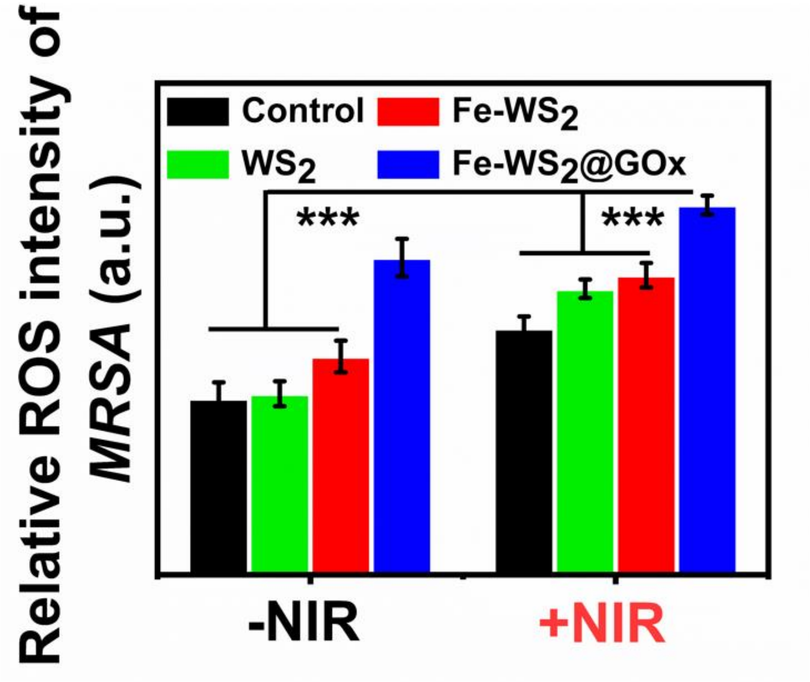

Figure S10. Detection of ROS content in MRSA after different treatments using DCFH-DA probe. N = 3. (*P ≤ 0.05, **P ≤ 0.01, ***P ≤ 0.001).

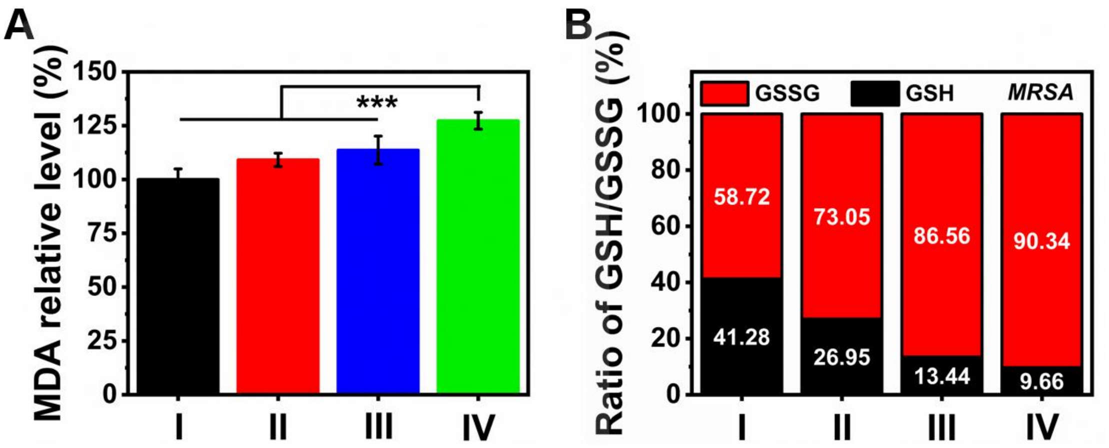

Figure S11. Detection of (A) MDA levels and (B) GSH/GSSG ratio within MRSA after different treatments. N = 3. (*P ≤ 0.05, **P ≤ 0.01, ***P ≤ 0.001). (I: Control; II: NIR; III: Fe-WS_2@GOx; IV: Fe-WS_2@GOx+NIR)

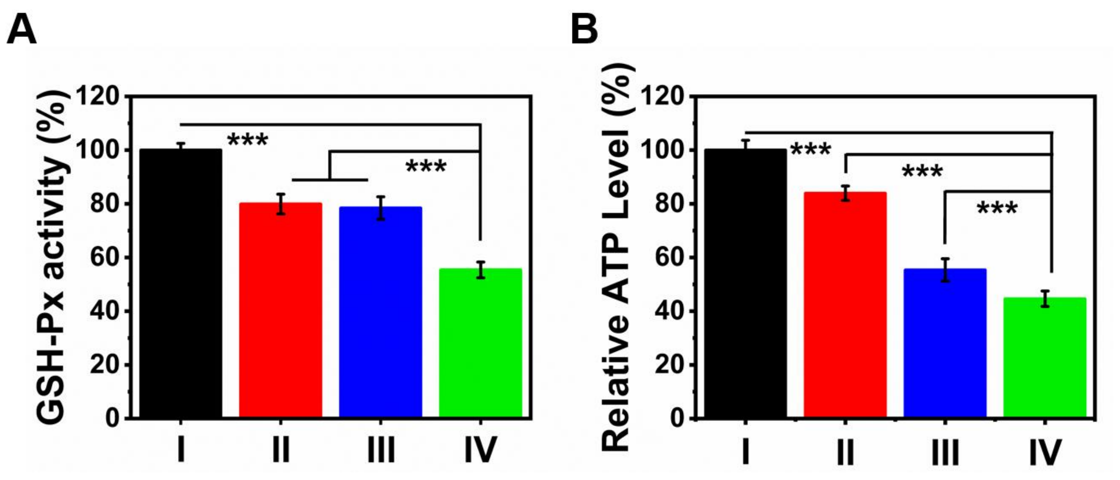

Figure S12. Detection of (A) GSH-Px and (B) ATP levels within MRSA by different treatments. N = 3. (*$P \leq 0.05$, **$P \leq 0.01$, ***$P \leq 0.001$). (I: Control; II: NIR; III: Fe-WS_2@GOx; IV: Fe-WS_2@GOx+NIR)

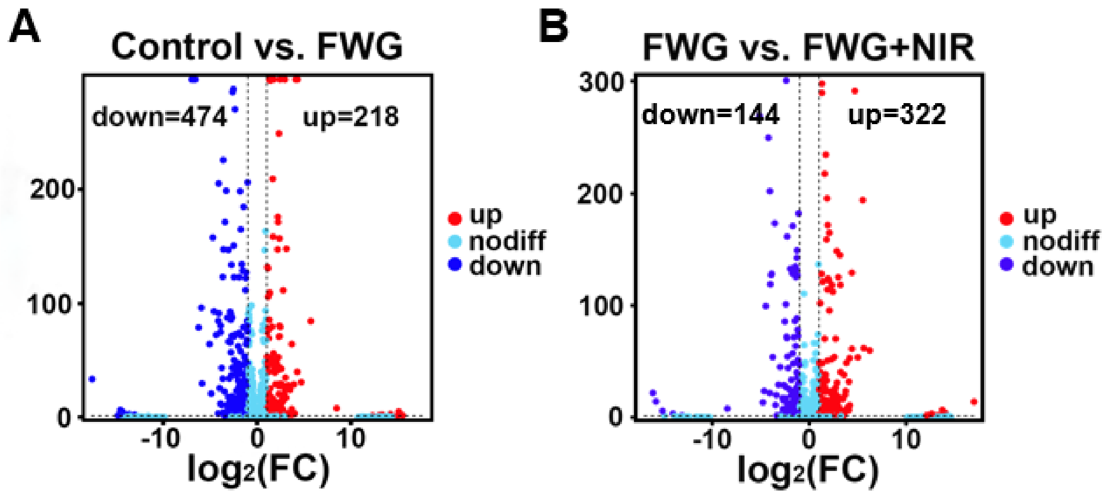

Figure S13. Volcano plots of up-regulated and down-regulated gene expression in (A) Control vs. FWG and (B) FWG vs. FWG+NIR groups. (FWG+NIR=Fe-WS_2@GOx+NIR; FWG= Fe-WS_2@GOx)

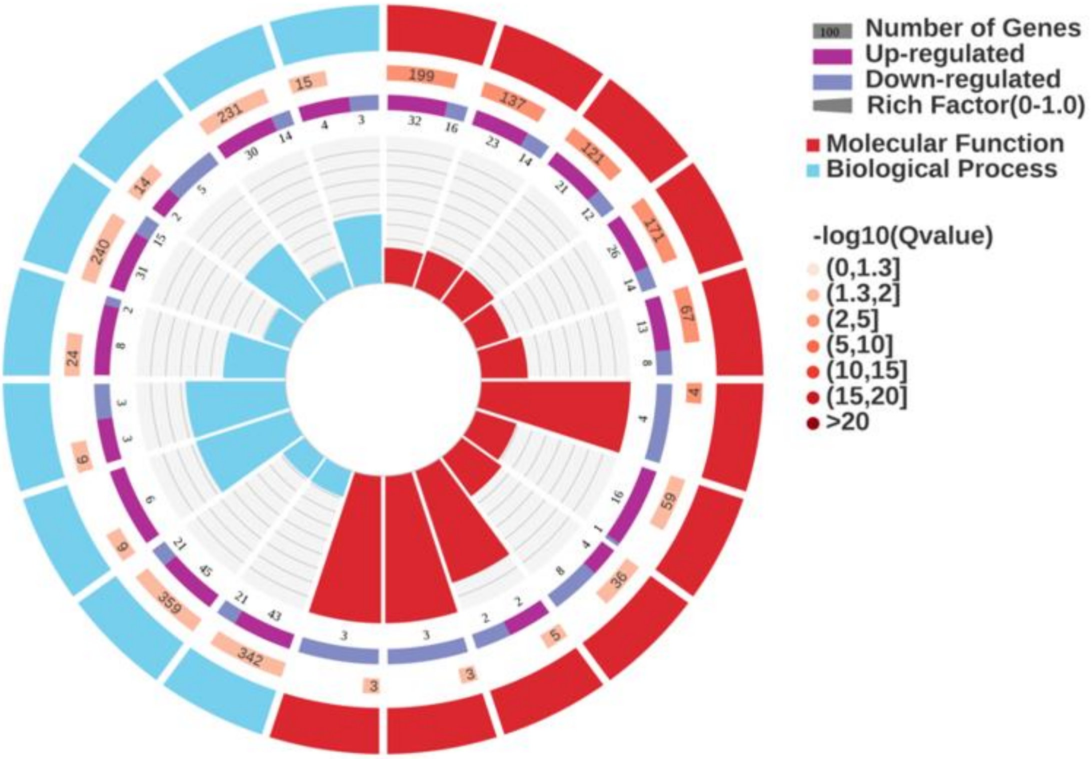

Figure S14. Differential expression results of genes analyzed by Fe-WS_2@GOx+NIR cyclic gene mapping.

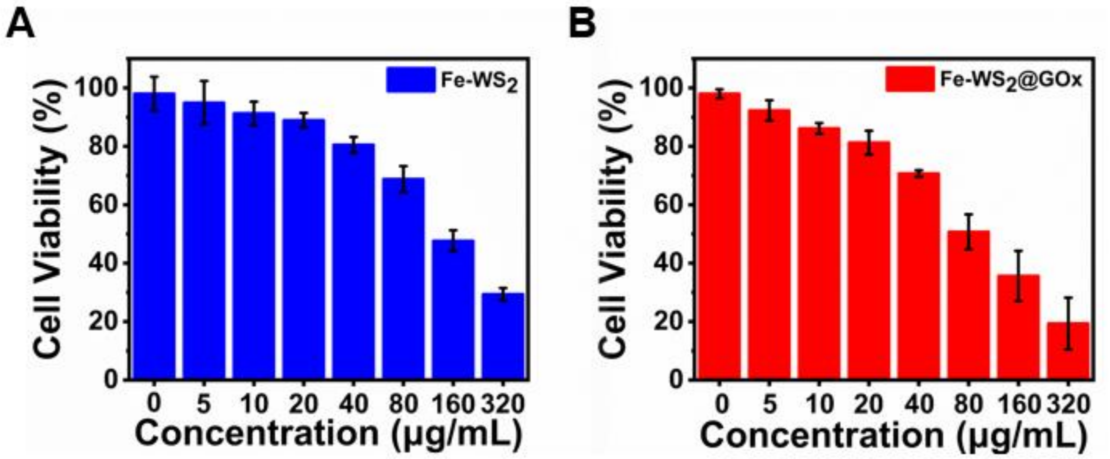

Figure S15. Cells viability of L929 assayed with different concentrations of (A) Fe-WS_2 and (B) Fe-WS_2@GOx. N = 3.

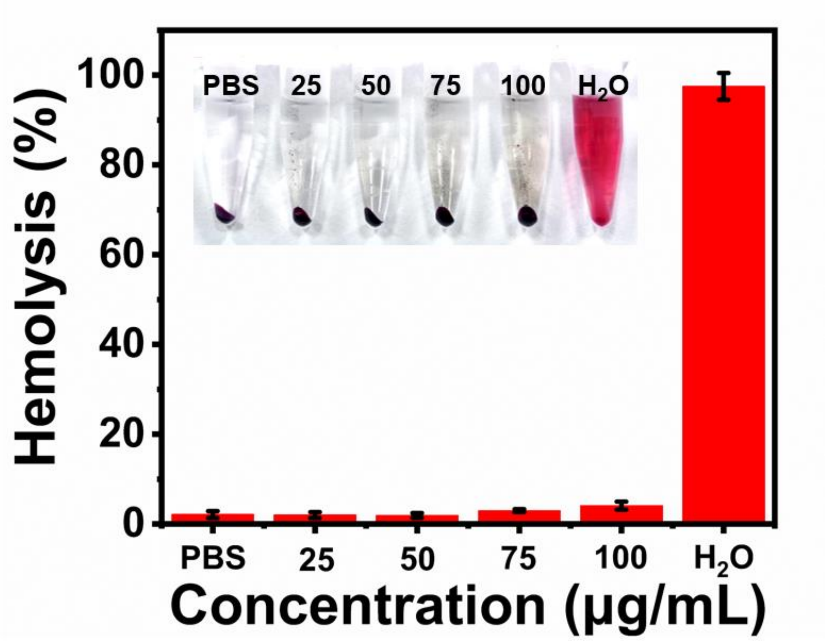

Figure S16. Hemolysis assay results after different treatments. N = 3.

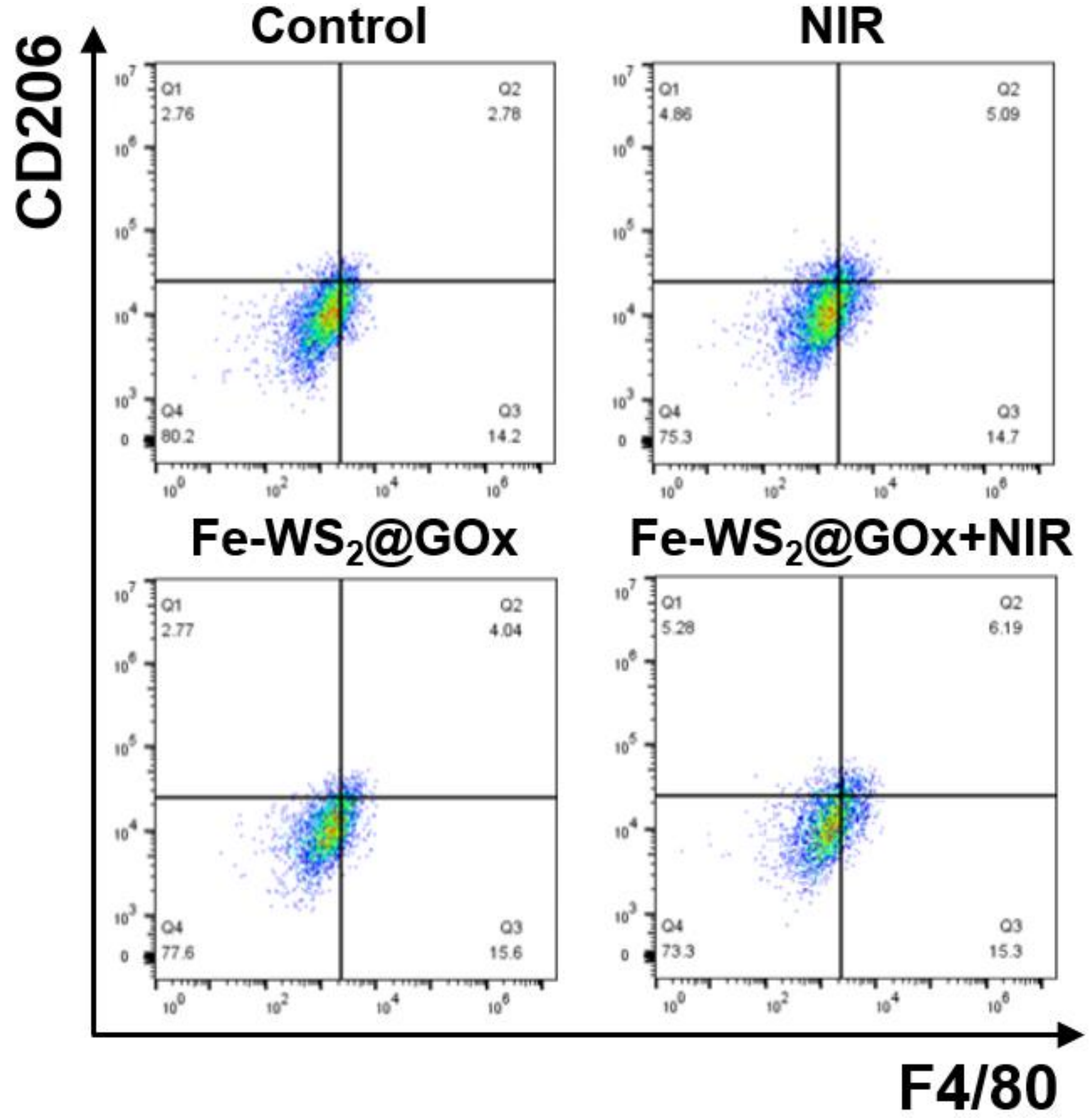

Figure S17. Macrophages were first polarized to M1 using LPS, and then the M2 phenotype of macrophages was detected using flow cytometry after different treatments.

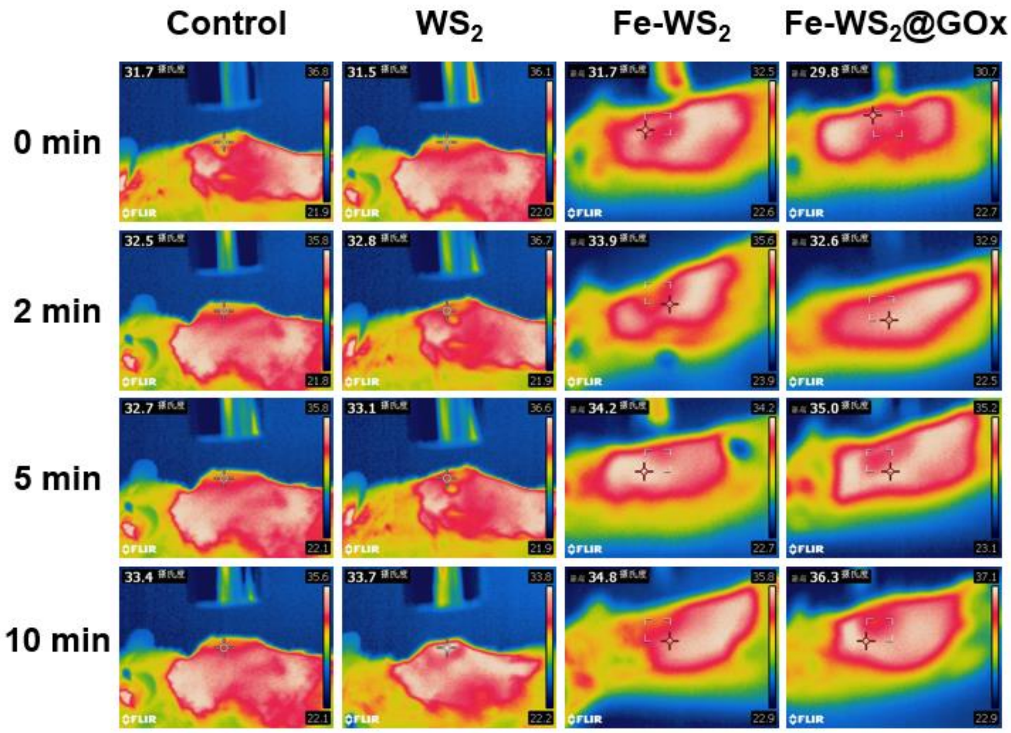

Figure S18. Mouse wounds were irradiated with 808 nm NIR (1.0 W/cm^2) for 10 min and temperature changes were recorded.

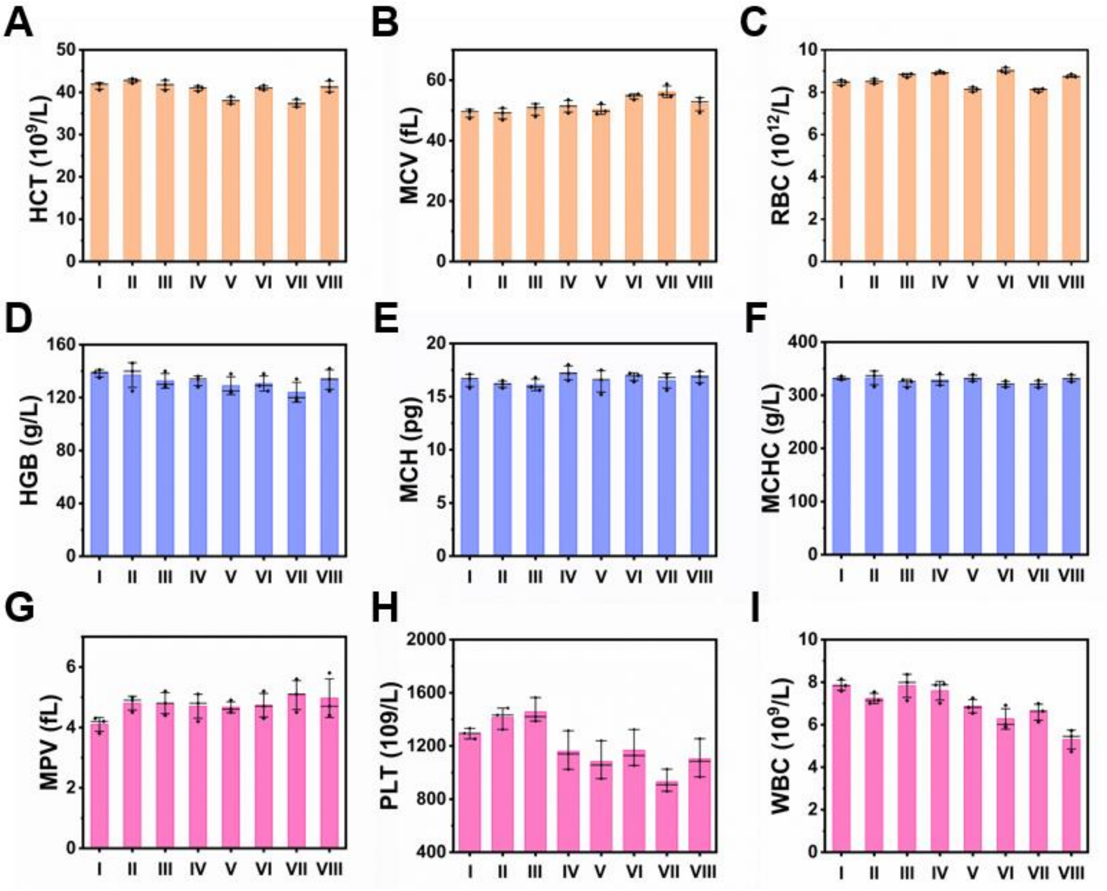

Figure S19. Blood routine test of mice in each group was tested. N = 3. (I: Control; II: WS_2; III: Fe-WS_2; IV: Fe-WS_2@GOx; V: NIR; VI: WS_2+NIR; VII: Fe-WS_2+NIR; VIII: Fe-WS_2@GOx+NIR)

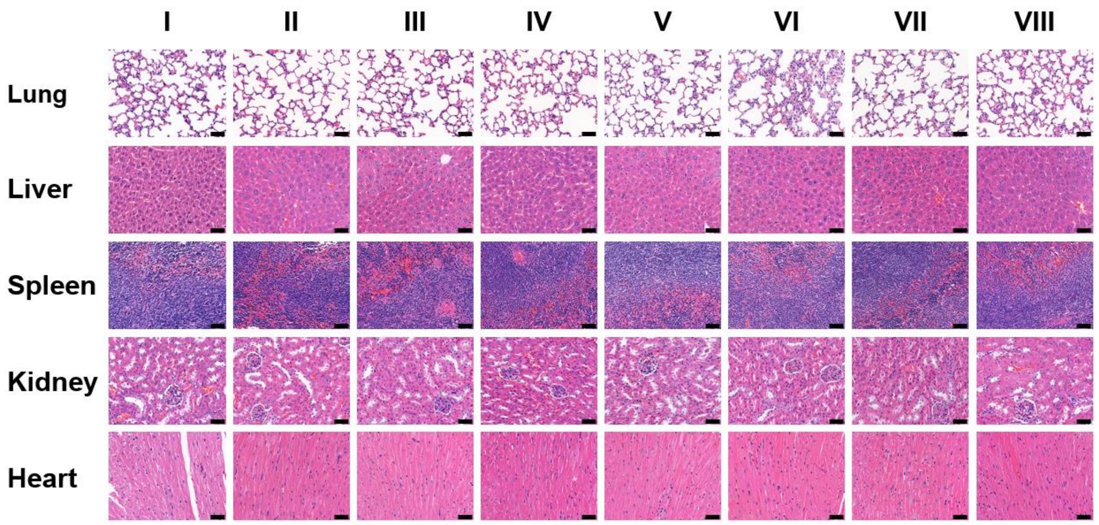

Figure S20. H&E staining of mice after different treatments. Scale bar = 50 μm. (I: Control; II: WS_2; III: Fe-WS_2; IV: Fe-WS_2@GOx; V: NIR; VI: WS_2+NIR; VII: Fe-WS_2+NIR; VIII: Fe-WS_2@GOx+NIR)
